# Highly Sustainable Plant and Yeast Hydrolysate-Based Alternative for Basal Medium Supports Long-Term *In Vitro* Propagation of Bovine Satellite Cells

**DOI:** 10.3390/foods15183207

**Published:** 2026-09-10

**Authors:** Lisa Schenzle, Shashank Goyal, Regina Leber, Harald Pichler, Aleksandra Fuchs

**Affiliations:** 1 acib—Austrian Centre of Industrial Biotechnology, 8010 Graz, Austria; lisa.schenzle@medunigraz.at (L.S.);; 2 EurA AG, 99084 Erfurt, Germany; shashank.goyal@eura-ag.de; 3 NAWI Graz, BioTechMed Graz, Institute of Molecular Biotechnology, Graz University of Technology, 8010 Graz, Austria; 4 Division of Physiology, Otto Loewi Research Center for Vascular Biology, Immunology and Inflammation, Medical University of Graz, 8010 Graz, Austria

**Keywords:** cultured meat, DMEM/F12, basal medium substitution, serum-free media, life cycle assessment, hydrolysate-based basal medium

## Abstract

The production of cultured meat will require large quantities of cell culture medium, presenting challenges related to high costs and limited sustainability of both protein-based components and other ingredients of the basal medium. To date, most research has focused on substituting and optimizing protein-based medium components, leading to significant progress in reducing costs associated with growth factors and serum albumin. However, the basal medium—containing amino acids, vitamins, carbon sources, and microelements—is equally essential. While some efforts have been made to optimize its composition, the environmental impact of using chemically fully defined mixtures remains considerable, and cost reductions are challenging. In this study, we introduce a novel approach to basal medium production by utilizing a mixture of partially hydrolyzed plant and yeast biomass. Our results demonstrate that hydrolysate-based basal medium (HBBM) performs comparably to chemically fully defined basal medium during proliferation and differentiation of bovine satellite cells. Furthermore, a comparative life cycle assessment was performed to explore the environmental impacts of replacing chemically fully defined medium with HBBM. The analysis showed potential reduction in environmental impacts, highlighting the relevance of basal medium formulation as a key element to influence ecological sustainability.

## 1. Introduction

Cellular agriculture has the potential to become an environmentally sustainable method of producing meat and seafood for human consumption; however, challenges related to production costs, safety, and high energy requirements still need to be addressed [1]. Much of this effect is still caused by the cell culture media costs. Classical cell culture medium typically consists of 5–20% fetal bovine serum (FBS), which includes growth factors, stabilizing agents, storage and adhesion proteins, in addition to a basal medium composed of carbon sources, amino acids, vitamins, and minerals, which usually has a chemically fully defined composition [2]. Substitution of FBS was a key focus of research in cellular agriculture in the last decade, leading to serum-free media formulations for isolation, cryopreservation, proliferation, and differentiation media for both cultured meat and seafood cell lines [2], additionally focusing on the possibilities to substitute all animal-sourced components with animal-free alternatives [3,4,5,6,7]. It also led to realization that the environmental [8], ethical and financial [3,9] advantages of using FBS-free cell culture are beyond reasonable doubt.

However, the most frequently used basal media for cultured meat are DMEM/F12 for the terrestrial cell lines and Ham’s F10 and DMEM/F12 for aquatic species—both chemically fully defined. And although efforts were made to optimize basal medium content [10,11], the sourcing of the single components for chemically defined basal media has a profound impact on the price and environmental footprint of the final product [8], making reductions challenging.

In this study we explore a completely different approach of using a complex nutrient mixture sourced from plant and yeast hydrolysates as a basal medium.

Hydrolysates obtained from plants or microorganisms using enzymatic or acidic digestion have been discussed as a cell culture component for several decades (see also the review in [12]). However, most of the effort was directed at using hydrolysates as a substitution for FBS, especially as hydrolysates from plant materials were effective in substituting serum for pharmaceutically relevant cell lines, such as CHO, HEK, VERO, and BHK, as well as insect cell lines [13,14,15]. Unfortunately, this approach did not show the same efficiency for cultured meat- or cultured seafood-relevant cell lines, allowing only partial substitution of FBS [16,17,18], although recently, plant-sourced [4,5] and microbial-sourced [6] alternatives to human serum albumin were shown effective for cultured meat-relevant cell lines.

Still, the nutrient-rich mixture of components, released via hydrolysis from plant or microbial biomass, can provide oligopeptides, free amino acids, vitamins and carbohydrates, minerals, and other bioactive components [19,20]. However, one has to take into account that the concentration of hydrolysates’ constituents can vary, as they are generally prone to batch-to-batch variations due to differences in hydrolyzation efficiencies and heterogeneous biomass content [21]. This could have a detrimental effect on cell growth, especially at scale.

Across Europe, wheat is one of the most traditionally cultivated and thus widely available plants, with rice and soy, the latter of which is most protein-rich of the three, swiftly gaining in importance in recent decades [22,23]. Diverse hydrolysates derived from these plants are cost-effective, relatively straightforward to produce, and already utilized in the fields of biotechnology, cosmetics, and nutrition [2,15,19,24,25]. An additional sustainability advantage could be achieved in future by using hydrolysates from plant-based agro-industrial waste, such as soybean meal, peanut meal, sunflower bran, press cakes, meals, rice bran, waste potato juice, etc. [18,26], which should also be considered as viable sources of ingredients for culture media to support the principles of a circular economy.

Moreover, hydrolysates obtained from agro-industrial by-products such as Brewer’s spent yeast have the potential to serve as sustainable and cost-effective sources of nutrients in basal media for the cultured food sector. Spent yeast hydrolysate contains up to 60% protein, with 40% of the total amino acids being composed of essential amino acids. Furthermore, it is rich in vitamins and minerals and abundant locally throughout many countries [27,28]. Such application would provide opportunities to enhance the value of waste and mitigate its adverse environmental impacts [29,30]. Whilst the majority of residual biomass from large-scale breweries is already subjected to a cascading use in the food and feed industries, smaller-scale breweries frequently dispose of their spent yeast, which significantly contributes to the organic load of wastewater [31]. Also, other yeast species’ waste biomass can be used, e.g., *Pichia pastoris* (i.e., *Komagataella phaffii*), which is increasingly used in precision fermentation and is gaining Generally Recognized as Safe (GRAS) status and Qualified Presumption of Safety (QPS) status for its products from the US Food and Drug Administration (FDA) and European Food Safety Authority (EFSA) respectively [32,33]. The integration of such local waste into the production of highly valuable components for culture medium could provide a solution to this problem.

Life cycle assessments (LCAs) also consistently identify culture media as a dominant environmental hotspot in cultured meat (CM) production due to the energy- and resource-intensive production of highly refined inputs [1,34]. Key contributors include amino acids, recombinant proteins including growth factors, vitamins, and carbon sources, whose manufacture relies on complex biochemical processes, extensive raw material extraction, and high energy demand. Amino acids alone can account for a substantial share of the culture medium’s carbon footprint, while recombinant proteins and growth factors exhibit highly variable impacts depending on production scale and energy source [1,35]. Additional environmental burdens arise from purification and processing steps, which are often insufficiently represented in current LCAs [36]. Furthermore, the frequent exclusion of media processing, waste handling, and spent media treatment leads to conservative impact estimates [37]. Improving the sustainability of CM, therefore, critically depends on culture medium reformulation, reduced refinement, and industrial-scale, low-carbon production of media components [38].

We thus hypothesize that hydrolysates can be used as a complete substitution for chemically fully defined basal media. In this study, we evaluated the possibility of such substitution of a chemically fully defined basal medium (e.g., DMEM/F12, widely used in cellular agriculture) with plant-derived and yeast-derived hydrolysates, while keeping other supplements constant, and to assess such substitution regarding scalability, costs, batch-to-batch variability and impurities. We have demonstrated that a plant and yeast hydrolysate-based basal medium (HBBM) performed comparably to DMEM/F12 in both short- and long-term proliferation experiments of bovine satellite cells. The substitution does not have a detrimental effect on differentiation capacity over the initial four passages. The analysis of HBBM indicates that the concentrations of individual components are significantly lower than those given for DMEM/F12, posing a future question of nutritional bottlenecks in high-cell-density environments. We have further conducted a comparative LCA to explore the ecological benefits of HBBM in comparison to DMEM/F12.

## 2. Materials and Methods

### 2.1. Materials

B8 (HiDef-B8 500X) was purchased from Defined Bioscience (#LSS-201, San Diego, CA, USA). *Saccharomyces cerevisiae* was acquired from the brewery Sudhaus Graz, and the CBS7435 *ku70-his4-HAC1* strain of *Pichia pastoris* was obtained from the strain collection of the TU Graz, Institute of Molecular Biotechnology. Wheat, rice and soy hydrolysates were developed and produced by the company NaTurtle using a proprietary process based on a previously published protocol [39]. Hydrolysates were provided in powder form, stored at room temperature, reconstituted in double-distilled water and sterile-filtered prior to application in cell culture.

### 2.2. Analysis of Hydrolysates

Analysis of hydrolysates and waste cell culture medium was performed by Eurofins Spinnovation Analytical B.V. (Oss, The Netherlands). Single measurements with no replicates of the listed components were performed. However, the analysis of amino acids routinely includes cross-checking the results from two orthogonal analytical methods, i.e., quantitative NMR and LC-MS/MS, generally resulting in within-batch Relative Standard Deviation (RSD) of about 1–8%. Exact numbers depend on the type and concentration of amino acid and on the complexity of the sample matrix.

### 2.3. Yeast Cell Extracts’ Preparation

*S. cerevisiae* cells were either cooled (at 4 °C) or prepared immediately upon sample receipt. *Pichia pastoris* cells were cultivated overnight in 50 mL Yeast–Peptone–Dextrose (YPD) broth, at 130 rpm and 28 °C, in a 250 mL baffled shake flask. Fresh biomass was washed 3 times with sterile double-distilled water (ddH_2_O, #16.231 Fresenius Kabi Austria GmbH, Graz, Austria) and centrifuged 3 times at 15,000 rpm to remove residual medium. Cell wet weight (10 g CWW) was resuspended in 25 mL sterile ddH_2_O and disrupted mechanically using 0.25–0.5 mm glass beads (#A553.1 Carl Roth, Karlsruhe-Mühlburg, Deutschland) with intermittent CO_2_ cooling. Cells were disrupted for 3 min in total, with 5 s CO_2_ cooling every 30 s. Cell debris and non-lysed cells were separated via centrifugation (3 times at 15,000 rpm), and the supernatant was harvested and stored at −80 °C. Prior to application in cell culture, the lysates were sterile-filtered using a 0.22 μm filter.

### 2.4. Yeast Autolysate Preparation

*S. cerevisiae* cells were either cooled at 4 °C or prepared immediately upon sample receipt. *Pichia pastoris* cells were cultivated overnight in 50 mL YPD at 130 rpm and 28 °C in a 250 mL baffled shake flask. Fresh biomass was washed 3 times with sterile ddH_2_O and centrifuged 3 times at 15,000 rpm. Cell wet weight (5 g CWW) was resuspended in 50 mL sterile ddH_2_O and transferred into a 250 mL baffled shake flask. Autolysis was carried out in a 50 °C incubator with shaking at 130 rpm (shake plate) for 50 h. Cell debris and non-lysed cells were separated via centrifugation (3 times at 15,000 rpm), and resulting supernatant (autolysate) was harvested and stored at −80 °C. Prior to application in cell culture, the lysates were sterile-filtered using a 0.22 μm filter.

### 2.5. Isolation of Satellite Cells

Bovine Satellite Cells (BSC) were isolated from three freshly sacrificed 19-month-old, castrated Simmental Oxen (*Bos taurus*) provided by Marcher Fleischwerke GmbH. Muscle tissue from *M. semitendinosus* was used, and the standard protocol described by Stout et al. [40] was applied. After isolation and following the initial growth phase, cells were cryopreserved in 20% FBS (#26140079 Thermo Fisher, Waltham, MA USA) with 10% dimethyl sulfoxide (DMSO #D2650 Merck, Darmstadt, Germany) or passaged for downstream screening or differentiation using 0.25% phenol-red trypsin-EDTA (#25200056 Thermo Fisher, Waltham, MA USA). All presented data was performed using BSCs from the same animal; cell pooling from different animals was not performed. Experiments in other two animals were performed as repetitions of the short- and long-term experiments presented here and displayed the same trends.

### 2.6. Confirmation of BSCs’ Identity and Differentiation

Following isolation, undifferentiated BSCs were identified by immunostaining for the Paired-box 7 (Pax7) marker as was previously shown [3]. BSCs were cultured on coverslips in BSC growth medium (GM) until they reached 70% confluence. Cells were fixed with 4% paraformaldehyde (#AAJ61899AK Fisher Scientific, Pittsburgh, PA, USA) for 30 min, washed with Dulbecco’s Phosphate-Buffered Saline (DPBS), and permeabilized for 15 min with 0.5% Triton-X (#T8787 Merck, Darmstadt, Germany) in DPBS. Cells were then washed with DPBS containing 0.1% Tween-20 (#P1379 Merck, Darmstadt, Germany). Primary Pax7 antibodies (#PA5-68506 Thermo Fisher, Waltham, MA USA) were diluted 1:500 in blocking solution (Antibody Diluent #S202230-2 Agilent Technologies, Santa Clara, CA, USA) containing 1:100 Phalloidin 594 (#A12381 Thermo Fisher, Waltham, MA USA) and applied for cell incubation at 4 °C overnight. Further, cells were washed with DPBS + Tween-20 and incubated for 1 h at room temperature with secondary antibodies for Pax7 (1:500, #A-11008 Thermo Fisher, Waltham, MA USA) and DAPI solution (1:1000). After final washes with DPBS + Tween-20, samples were mounted using ProLong Glass Antifade Mountant (#P36984, Thermo Fisher, Waltham, MA USA). Visualization and imaging were performed using a ZEISS Axio Imager 2 microscope (Oberkochen, Germany) to confirm the purity of the isolated satellite population, as shown previously in Supplementary Figure 1 in [3].

Isolated BSCs were differentiated for ten days. After growing cells to confluency in GM, the medium was changed to Differentiation Medium (DM) and then 50% of the medium was regularly changed (every two to three days) until BSCs reached differentiated state and were fixed and prepared as previously described. Desmin (#D1033-100 UL Merck, Darmstadt, Germany) was diluted 1:100 in blocking solution (Antibody Diluent #S202230-2 Agilent Technologies, Santa Clara, CA, USA) and incubated overnight at 4 °C. A secondary antibody (#A-11001 Thermo Fisher, Waltham, MA USA) was diluted in blocking solution 1:1000 with DAPI solution (1:1000) and incubated at room temperature for 1 h. ProLong Glass Antifade Mountant (#P36984, Thermo Fisher, Waltham, MA USA) was then applied. For determination of the fusion index, desmin-stained cells were used, and the number of DAPI-stained nuclei was quantified using the ZEISS ZEN cell-counting module, as described in Supplementary Figure 1 in [3]. After that, cells containing more than 2 nuclei per cell were manually counted [41]. A range of 150–250 nuclei was counted for each image, with three images processed per sample.

### 2.7. Media Composition

Skeletal muscle tissue samples were transferred into a transport medium consisting of DMEM/F-12 (#11320033 Thermo Fisher, Waltham, MA USA) supplemented with 1% Antibiotic–Antimycotic (#15240062 Thermo Fisher, Waltham, MA USA) immediately after extraction. For isolation of BSCs, Primocin growth medium (P-GM) was used, containing DMEM/F-12, 1% Primocin (Invitrogen #ant-pm-1), 20% fetal bovine serum (FBS; #26140079 Thermo Fisher, Waltham, MA USA) and 1 ng/mL human FGF basic/FGF2/bFGF (#233-FB-025/CF R&D Systems, Minneapolis, MN, USA). After the first passage, P-GM was changed to growth medium (GM), in which Primocin was substituted with 1% Antibiotic–Antimycotic (#15240062 Thermo Fisher, Waltham, MA USA).

For described short-term and long-term experiments, 96-well (#655180 Greiner Bio-One, Kremsmünster, Austria) or 6-well tissue culture plates (#657160 Greiner Bio-One, Kremsmünster, Austria) were used with GM and 1.5 µg/cm^2^ recombinant human Vitronectin (#15134499 Fisher Scientific, Pittsburgh, PA, USA). After an initial 24 h, cells were washed with DPBS, and B8 medium was added, which consisted of DMEM/F12 supplemented with HiDef-B8 (#LSS-201 Defined Bioscience, San Diego, CA, USA) as well as 1% Antibiotic–Antimycotic. B9 medium contains B8 medium supplemented with 0.8 g/L human serum albumin (HSA, #A9731 Merck, Darmstadt, Germany). Either DMEM/F12 or the designated hydrolysates were used as basal medium. B9 + MC medium additionally contains 0.1 g/L methyl cellulose (MC) (#M0512 Merck, Darmstadt, Germany) as a growth factor-stabilizing agent [3].

For basal medium substitution experiments (DMEM/F12 was substituted with plant/yeast hydrolysates), HiDef-B8 medium aliquots were transferred into DPBS supplemented with 1% Antibiotic–Antimycotic and 0.8 g/L HSA. Further, for yeast cell extracts, autolysates and plant hydrolysates, total protein concentration was measured using a BCA assay to determine protein and peptide content (amino acid number > 3), and designated dilutions in DPBS were tested for DMEM/F12 substitution as described below.

### 2.8. Screening of BSCs

BSCs were thawed in 5 mL GM and centrifuged at 200× *g* for 3 min. The supernatant was discarded, and the cells were washed with 5 mL DPBS. Both short- and long-term experiments were conducted as previously described [3].

### 2.9. Presto Blue Assay

To measure the metabolic activity, which correlates with cell number, a Presto Blue assay was performed as previously described [3].

### 2.10. Hoechst Assay

To support the Presto Blue assay results, the Hoechst 33258 assay was used to quantify DNA per well, which directly correlates with the cell number. On the final screening day, plates were frozen at −80 °C. After thawing to room temperature, 100 µL ddH_2_O was added per well and the plates were incubated at 37 °C for 1 h. The plates were then frozen again at −80 °C and thawed to room temperature. Subsequently, 100 µL of Hoechst dye reagent—prepared by diluting 25 µL Hoechst 33258 stock solution (10 mg Hoechst 33258 (#H1398, Thermo Fisher, Waltham, MA USA) per mL in DMSO + ddH_2_O (1:4 *v*/*v*)) in 10 mL TNE buffer (10 mM Tris, 2 M NaCl, 1 mM EDTA, 2 mM sodium azide, pH 7.4)—was added to each well. After 15 min of incubation at room temperature, fluorescence was measured at 352 nm (excitation) and 461 nm (emission).

### 2.11. Statistical Analysis

For statistical analysis of Presto Blue and Hoechst assays, the data was tested for significance using ordinary one-way ANOVA followed by Dunnett’s multiple-comparison test using GraphPad Prism version 10.2.3. Statistical significance was defined as *p* < 0.05 (*), *p* < 0.01 (**), *p* < 0.001 (***) and *p* < 0.0001 (****). Non-significant results (*p* ≥ 0.05) were marked as “ns”. The error bars in the graphs represent the standard deviation.

### 2.12. Life Cycle Assessment (LCA)

A comparative LCA was conducted to evaluate the environmental impacts of two basal culture media used in cultured meat production: a conventional chemically fully defined medium (DMEM/F12, HEPES #01-170-1A, Sartorius, Göttingen, Germany) and an alternative formulation (HBBM). The goal was to assess relative differences in environmental performance attributable to medium formulation. The functional unit was defined as one liter of basal culture medium prepared for mammalian cell cultivation. A cradle-to-gate system boundary was applied, covering raw material extraction, production, and processing of culture medium components up to medium preparation. Downstream processes, including cell cultivation, spent media treatment, and end-of-life stages, were excluded.

Life cycle inventory data were compiled with the support of acib GmbH, established databases (such as Ecoinvent 3.11 and Agribalyse v3.1), and peer-reviewed literature, representing the production of amino acids, vitamins, minerals and other basal medium components. Identical background assumptions, including energy mix and production scale, were applied to both media to ensure comparability. For HBBM, the analysis focused on the impact of amino acids, vitamins, inorganic salts, organic acids, lipids and other compounds on the environment. Environmental impacts were assessed in terms of climate change, water use, land use, and resource use using a recognized life cycle impact assessment method (Environment Footprint 3.1) in accordance with ISO 14040/44 [42,43]. The software used for the analysis was LCA for Experts, version 10.9.6.1. Results are reported as relative differences between the two media formulations. The environmental impact values displayed in Section 3.10 are deterministic model outputs derived from the fixed compositions of HBBM and DMEM/F12 combined with background characterization factors, rather than means of replicate measurements; standard deviations are therefore not applicable. Propagating parameter/data-quality uncertainty from the background database was outside the scope of the present analysis and is noted as a direction for future work. The datasets used for the LCA analysis were based on the recommendation from Blackstone et al. [44], and are listed in the Supplementary Materials, Table S4.

## 3. Results

### 3.1. Production of High-Nutrient Cell Lysates and Hydrolysates from Plant and Yeast Biomass

**Figure 1:**
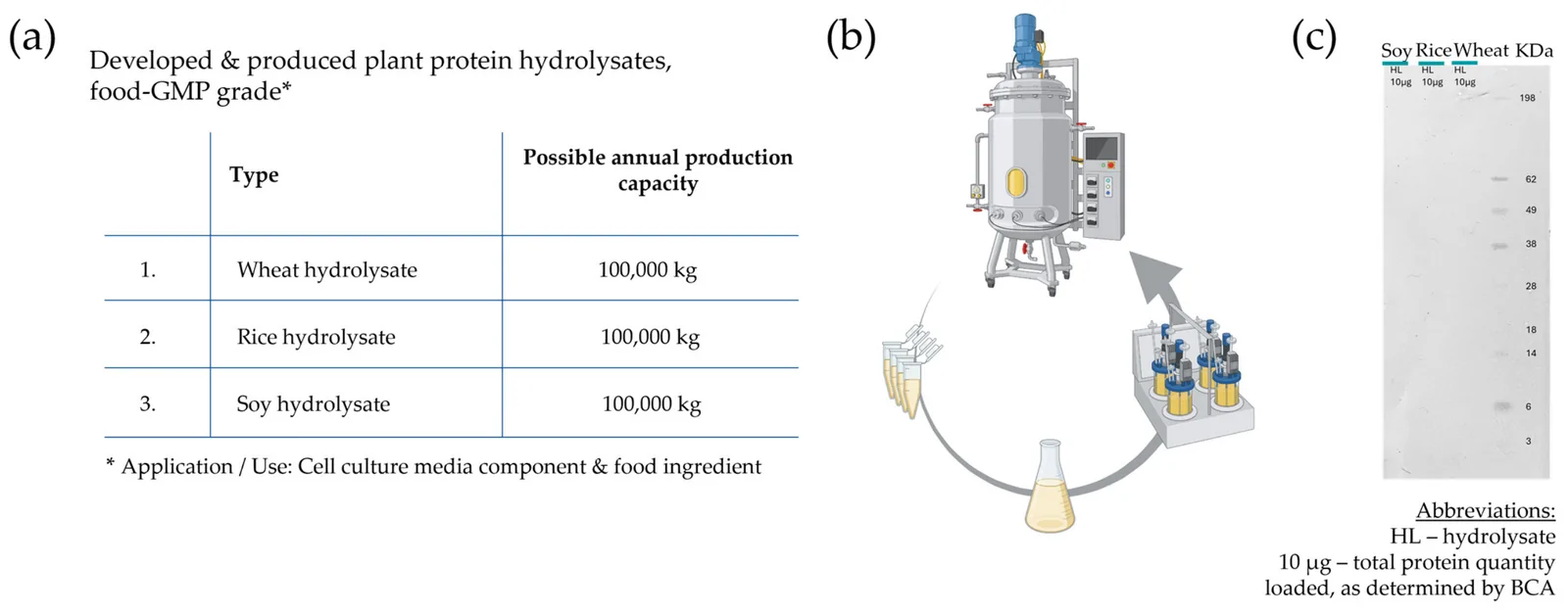
NaTurtle developed protein hydrolysates from conventional food plants—wheat, rice and soy. (**a**) List of the plants used and possible annual production capacity using a proprietary process, schematically depicted in (**b**). (**c**) Effective proteolysis to peptides and amino acids was demonstrated via SDS-PAGE; 10 μg protein were proteolysed, separated by SDS-PAGE and stained with Coomassie blue. (**b**) was created in BioRender. Fuchs, A. (2026) https://BioRender.com/zasfzpp (accessed on 6 September 2026).

In collaboration with the company NaTurtle, several plant cell extracts and hydrolysates were produced from conventional food plants like wheat, rice, and soy (see Figure 1). For production of plant hydrolysates in powder form, a proprietary process was developed by NaTurtle, which is based on a published protocol [39] (Figure 1a,b). High hydrolysis efficiency was demonstrated for all three plant sources (Figure 1c).

Additionally, yeast extracts and autolysates were produced from two yeast species where one could reasonably expect a local left-over cell biomass (*Saccharomyces cerevisiae* from Sudhaus brewery Graz, Austria, as well as the CBS7435 *ku70-his4-HAC1* strain of *Pichia pastoris*). To produce yeast cell extract and autolysate, standard in-house protocols were applied (see Materials and Methods Section). Autolysis was performed without prior cell lysis as we assumed it would be more applicable for upscaling. Protein content of *S. cerevisiae*-based autolysate was reproducibly higher than that of *P. pastoris*-based autolysate (Figure S1a). This highlights the need for method optimization for different yeast species, as the applied method was originally developed for *S. cerevisiae*. The effectiveness of the autolysis for both species was demonstrated by the SDS-PAGE analysis, showing a high autolysis degree after 50 h (Figure S1b).

### 3.2. Short-Term Proliferation Tests of Hydrolysates on Proliferation of BSCs

Short-term proliferation assessments were conducted in serum-free medium spiked with lysates to identify those that consistently demonstrated an enhancing effect on cell proliferation beyond the levels observed in the control group—DMEM/F12. Such effects were also previously reported for diverse protein hydrolysates, where they were shown to improve cell viability and proliferation rate, e.g., for CHO, HEK, VERO, and BHK as well as for insect cell lines [13,14,15]. Through this screening process, effective working concentrations of the well-performing lysates were established.

**Figure 2:**
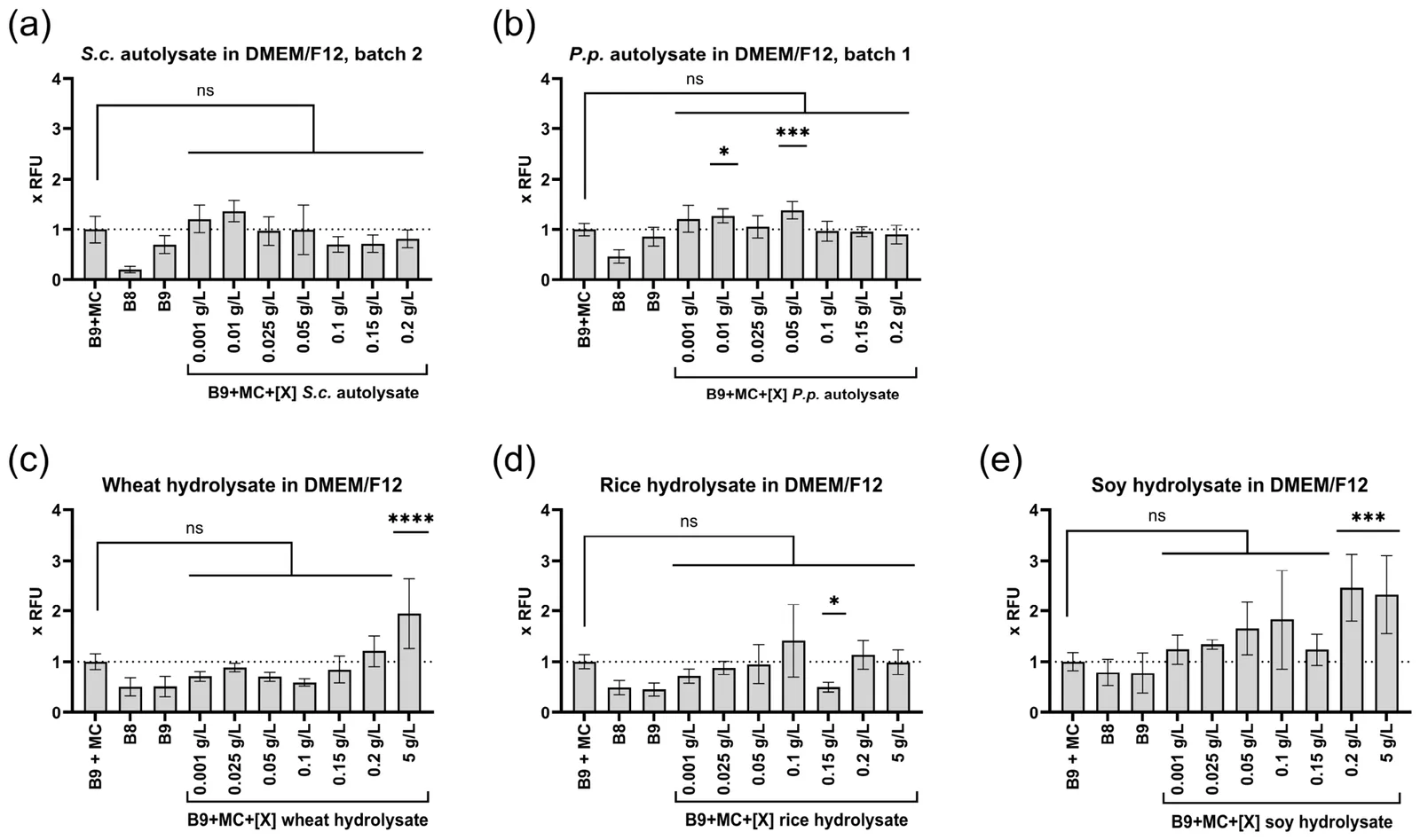
Short-term toxicity tests of the produced lysates in the serum-free medium B9 + MC, using BSCs as reporter cells; 2000 BSCs/cm^2^ were seeded on day 0 in GM and changed on day 1 to the designated medium. (**a**,**b**) Yeast autolysates and (**c**–**e**) plant hydrolysates in a range of concentrations were supplemented to the B9 + MC medium and, as indicated, a Hoechst assay (day 7) was performed to assess the viability and proliferation rate of the BSCs. Obtained values were normalized to BSCs in B9 + MC. Number of technical replicates, n = 6; the experiment was repeated twice; statistical significance was calculated by one-way ANOVA combined with Dunnett test, comparing all samples to B9 + MC, and is indicated by asterisks: *p* > 0.05 (ns), *p* < 0.05 (*), *p* < 0.001 (***), *p* < 0.0001 (****). Dashed line marks the level of the control. *P.p.*, *Pichia pastoris*; *S.c. Saccharomyces cerevisiae*; MC, methyl cellulose.

Insignificant toxicity was demonstrated for *S. cerevisiae* autolysates at concentrations above 0.025 g/L, with a slightly positive effect on proliferation for concentrations below 0.025 g/L (Figure 2a). *P. pastoris* autolysate had a significantly positive effect on proliferation of BSCs in the range of 0.01 to 0.05 g/L (Figure 2b). Wheat, rice and soy hydrolysates did not have any negative effects on the proliferation of BSCs and, at high concentrations—5 g/L in the case of wheat and 0.2 g/L and 5 g/L in the case of soy—exerted a significant proliferation-inducing effect (Figure 2c–e). These results were supported by the matching effects on proliferation timeline assessed using the Presto Blue Assay (see Figure S2).

### 3.3. Short-Term Tests of Full Substitution of DMEM/F12 by Yeast and Plant Hydrolysates

**Figure 3:**
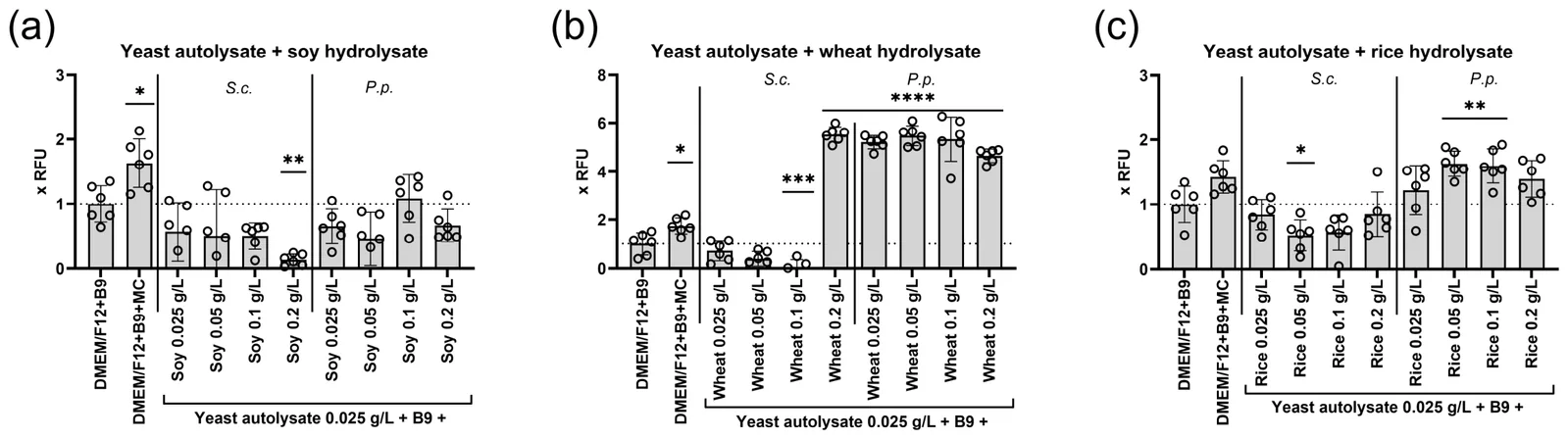
Full substitution of DMEM/F12 medium with plant and yeast hydrolysates; 2000 BSCs/cm^2^ were seeded on day 0 in GM and changed on day 1 to the designated medium. Here, 0.025 g/L yeast autolysate was chosen as the best working concentration, and plant hydrolysates of (**a**) soy, (**b**) wheat or (**c**) rice in a range of concentrations were supplemented to the B9 + MC add-on medium without DMEM/F12. Hoechst assay (day 7) was performed to assess the viability and proliferation rate of the BSCs. Obtained values were normalized to BSCs in DMEM/F12 + B9. Number of technical replicates, n = 6; the experiment was repeated twice. Statistical significance was calculated by one-way ANOVA combined with Dunnett test, comparing all samples to DMEM/F12 + B9, and is indicated by asterisks: *p* > 0.05 (not significant, not displayed), *p* < 0.05 (*), *p* < 0.01 (**), *p* < 0.001 (***), *p* < 0.0001 (****). Dashed line marks the level of the control. *P.p.*, *Pichia pastoris*; *S.c. Saccharomyces cerevisiae*; MC, methyl cellulose.

In all yeast and plant hydrolysates the pH was adjusted to 7.2. Glucose—the most commonly used carbon source for mammalian cells’ cultivation [45]—was absent in all yeast and plant hydrolysates tested. Thus, a dextrose concentration typically used in DMEM (17.5 mM) was supplemented to the hydrolysate-based basal medium (HBBM). B9 and B9 supplemented with growth factor-stabilizing methyl cellulose (B9 + MC), both containing DMEM/F12, were used as controls. However, despite glucose supplementation in serum-free medium and pH adjustments, a complete replacement of the basal medium with single cell extracts or hydrolysates—whether yeast- or plant-based—was not successful for BSCs (see Figure S3a–d). Thus, in the next step, the best performing plant- and yeast-hydrolysates at proliferation-promoting concentrations were combined to identify a formulation that could serve as a complete replacement for DMEM/F12 (Figure 3).

For this, 0.025 g/L yeast autolysate was chosen as the best working concentration, based on data presented in Figure 2. As demonstrated in Figure 3, in a short-term proliferation experiment with BSCs as reporter cells, a combination of 0.025 g/L of *P. pastoris* yeast autolysate and soy, wheat or rice hydrolysates performed well as a full DMEM/F12 substitute. Additionally (Figure S3e,f), both yeast autolysates were combined and tested as a full substitute of DMEM/F12; however, BSCs displayed affected growth and attachment deficits, rendering this combination unusable.

### 3.4. Long-Term Tests of Full Substitution of DMEM/F12 by Yeast and Plant Hydrolysates

**Figure 4:**
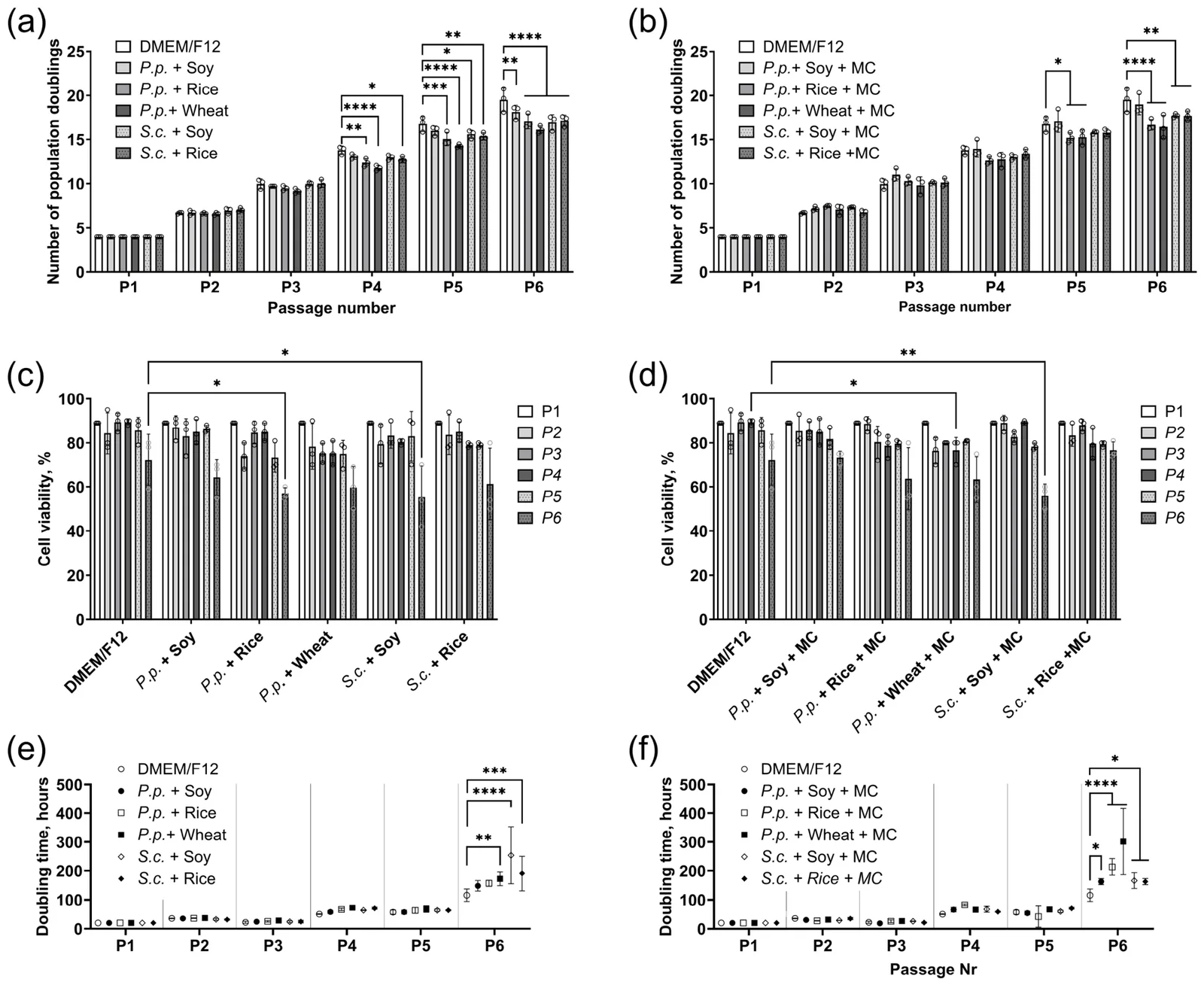
Long-term proliferation experiments using yeast and plant hydrolysates as a full substitution of DMEM/F12. BSCs cells were cultured in 6-well plates in B9 add-on media, using as basal medium either DMEM/F12 or a combination of 0.1 g/L plant hydrolysates combined with 0.025 g/L yeast autolysates named HBBM. Both hydrolysates were added immediately upon splitting, whereas HSA of the B9 add-on medium was added 24 h after splitting. Number of technical replicates was n = 3; the experiment was repeated twice. Average population doublings and doubling time for passage 1 were assessed based on cell count upon isolation of BSCs. During passaging, trypan blue was used to assess viability and cells were counted using Invitrogen Cell Countess. Resulting number of viable cells (**c**,**d**) was used to calculate population doublings (**a**,**b**) and doubling time (**e**,**f**). Statistical significance was calculated by two-way ANOVA combined with Dunnett’s multiple-comparison test, comparing all samples to DMEM/F12, and is indicated by asterisks: *p* > 0.05 (not significant, not displayed), *p* < 0.05 (*), *p* < 0.01 (**), *p* < 0.001 (***), *p* < 0.0001 (****). *P.p.*, *Pichia pastoris*; *S.c. Saccharomyces cerevisiae*; MC, methyl cellulose.

The best combinations from the short-term experiments were further validated in long-term proliferation experiments over six passages (Figure 4). Here, both MC-supplemented and MC-free media were evaluated, as the effect of this low-cost growth factor and cell stabilizer [3], when combined with alternative basal media, was unknown in the multi-passage set-up.

One of the hydrolysate combinations performed comparably to DMEM/F12, although not reaching its efficiency in the long run (*P. pastoris* + soy, see Figure 4a,c,e). Its performance became slightly improved by the addition of MC (Figure 4b,d,f), but still not quite reaching the level of DMEM/F12. *S. cerevisiae* hydrolysate generally performed worse than *P. pastoris* hydrolysate, confirming the outcome of the short-term experiments (Figure 3), with the combinations rice–*S. cerevisiae* and soy–*S. cerevisiae* yielding similar outcomes (Figure 4). Additionally, differentiation efficiency of the BSCs in *S. cerevisiae*-based basal medium was visibly lower than in DMEM/F12, whereas morphology of BSCs grown and differentiated in *P. pastoris* hydrolysate-based medium was more similar to those in DMEM/F12, as we demonstrate in Figure S4.

We have further compared the morphology of non-differentiated BSCs in *P. pastoris* hydrolysate combined with soy, rice or *S. cerevisiae* hydrolysates. Cells on day 4 were examined and no significant difference in morphology was detected, although in both inspected plant hydrolysate-containing samples, the rounded-up, semi-detached cells were more often present (Figure S5a,b), which might imply adhesion issues. Cell counts additionally supported this hypothesis, as it was significantly lower in plant-hydrolysate-containing media, as compared to control or only yeast-hydrolysate-containing medium (Figure S5d). However, MC supplementation alleviated this effect in soy-hydrolysate-containing medium (Figure S5c).

### 3.5. Timing of the Plant Hydrolysates’ Addition Impacts Viability of BSCs in HBBM

Since the short-term performance of BSCs with certain combined lysates surpassed proliferation levels observed in DMEM/12-containing media (Figure 3), we concluded that a better performance of HBBM in long-term experiments can be expected. Thus, the protocol for long-term experiments with these lysates was further optimized. As the most potent area for improvement, we have chosen the adhesion issues observed in plant-hydrolysate-containing media, as described in Section 3.4. These issues may be attributed to the presence of plant albumins or their partially hydrolyzed proteins, possessing coating abilities.

For example, plant seeds contain high quantities of albumin-like molecules [46,47,48,49]. Such plant albumins were previously shown to constitute a big soluble fraction of the seeds’ whole protein isolations [4,5]. And due to similar functionality, they might affect the adhesion capacity of the adherent BSCs—even upon hydrolysis—when supplemented immediately during plating. The same effect was demonstrated for non-hydrolyzed Human Serum Albumins (HSA) [9]. We thus hypothesized that protein hydrolysates could still contain peptides possessing blocking capacity. To test this hypothesis, a long-term proliferation experiment was performed with the supplementation of plant hydrolysates 24 h after every splitting.

**Figure 5:**
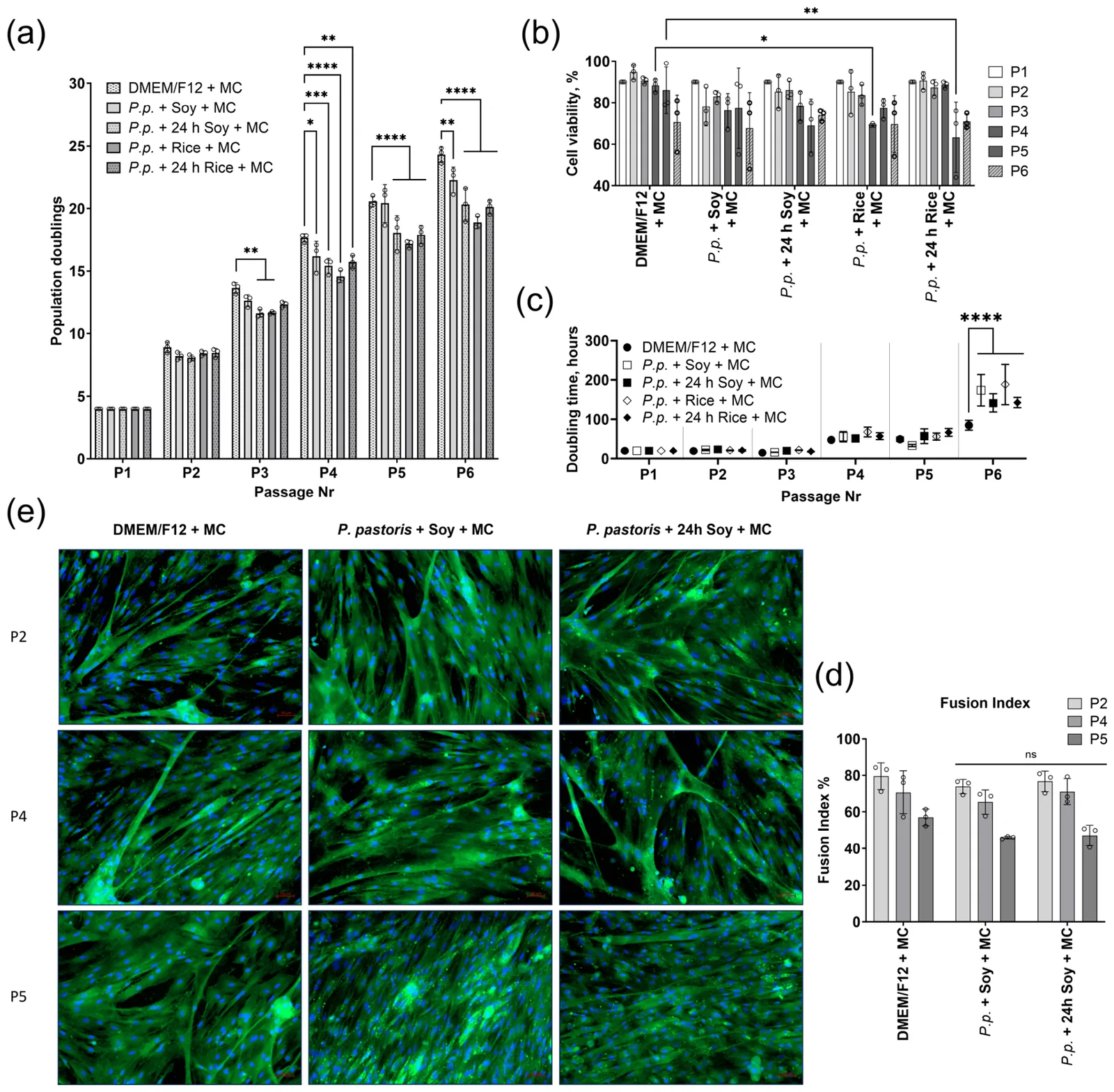
A 24 h delay in addition of plant hydrolysates during long-term proliferation in yeast- and plant-based basal medium. BSCs cells were cultured in 6-well plates in B9 add-on media, using either DMEM/F12 or a combination of 0.1 g/L soy hydrolysate and 0.025 g/L *P.p.* autolysate as basal medium. Plant hydrolysates were added either immediately upon splitting or, where stated, 24 h later. HSA (as a part of B9 add-on medium) was added 24 h after splitting. Number of technical replicates was n = 3, and experiment was repeated twice. Average population doublings and doubling time for passage 1 were assessed during isolation of BSCs. During passaging, viable cells were counted as described in the Materials and Methods Section, and the number of viable cells (**b**) was used to calculate population doublings (**a**) and doubling time (**c**). During passaging, a portion of the cells were seeded for differentiation and IF staining (**e**), and fusion indices (**d**) were calculated as a measure of differentiation capacity. For the fusion indices, the ratio of cells with at least two nuclei to all nuclei was used. A range of 150–250 nuclei was counted for each image, with three images processed per sample. The DAPI count was performed by the Zeiss Zen tool for cell counting. Immunofluorescence staining was performed for nuclei (DAPI, blue) and desmin (green). Scale bar = 100 μm. Microscope Zeis Axio Imager. Lens: EC Plan-Neofluar 10×; DAPI exposure: 20 ms. *P.p.*, *Pichia pastoris*; *S.c. Saccharomyces cerevisiae*; MC, methyl cellulose. *p* > 0.05 (ns), *p* < 0.05 (*), *p* < 0.01 (**), *p* < 0.001 (***), *p* < 0.0001 (****).

This strategy had a significantly positive effect on cell viability—for soy and especially for rice hydrolysates (Figure 5b). In case of rice hydrolysate, this effect led to higher population doublings, but not in case of soy (Figure 5a). Cell morphology upon differentiation (Figure 5e) and differentiation capacity within the first four passages, as assessed via fusion index (Figure 5d), were unchanged in the samples grown in fully substituted HBBM, as compared to DMEM/F12.

We thus concluded that plant albumin peptides or other constituents, present in plant hydrolysates, might have indeed affected cell adhesion. Nonetheless, a 24 h delay in the addition of plant hydrolysates to cell culture media may be detrimental to cell viability, and additional efforts should be undertaken to minimize the delay in supplementing plant hydrolysates as part of HBBM.

### 3.6. Content Analysis of the Hydrolysate-Based Full Substitute of DMEM/F12

Further, *P. pastoris* and *S. cerevisiae* autolysate and soy hydrolysate compositions of amino acids, vitamins, sugars and minerals were analyzed and compared to DMEM/F12. Possible bottlenecks in amino acid compositions were identified, based on the list of bovine essential amino acids (marked with red E) [50].

### 3.7. Analysis of the Free Amino Acid Content of the Hydrolysates

We show that batch-to-batch variations are quite substantial (approx. 30–40%) after the laboratory-scale autohydrolysis of the *S. cerevisiae* yeast samples (Figure S6), demonstrating the need for method optimization and standardization.

**Figure 6:**
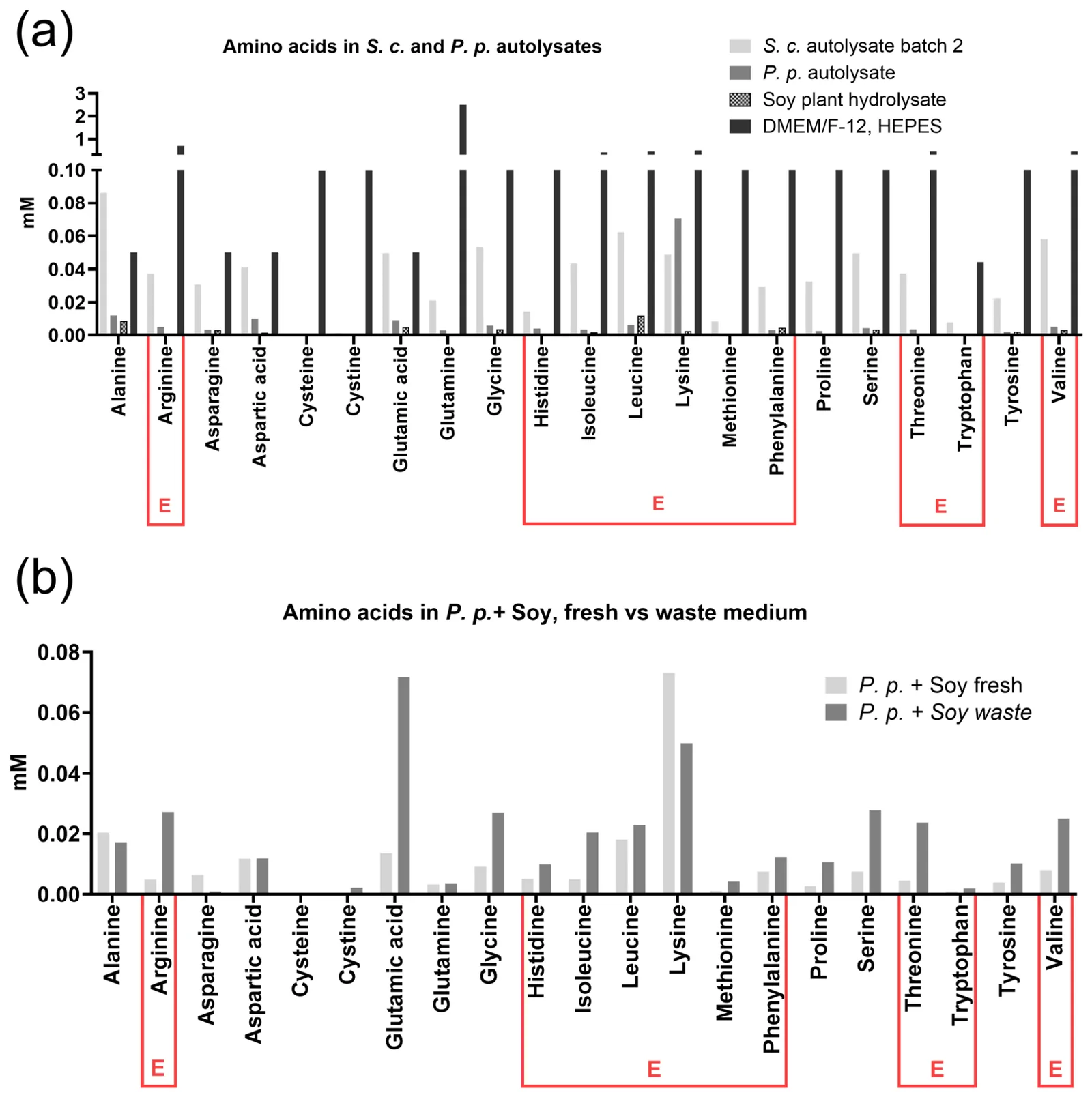
Amino acid content of the yeast and plant hydrolysates. Analyzed concentrations were adapted to reflect the best working concentrations in the cell culture media (0.1 g/L soy hydrolysate combined with 0.025 g/L *P.p.* autolysate). Essential bovine amino acids are marked with red “E”. (**a**) Comparison of several yeast autolysate preparations of *Saccharomyces cerevisiae* from Sudhaus Graz and in-house strain of *Pichia pastoris*. DMEM/F12 contents are plotted as a control. (**b**) Comparison of the amino acid content in fresh HBBM and waste HBBM combined with B9 add-on media after 3 days of BSC proliferation, passage 3. *P.p.*, *Pichia pastoris*; *S.c.*, *Saccharomyces cerevisiae*.

Amino acid concentrations of the best working combination (0.1 g/L soy hydrolysate combined with 0.025 g/L *P. pastoris* autolysate) of HBBM appeared in general to be much lower than that used in DMEM/F12 composition (Figure 6a).

As shown by our analysis, all essential bovine amino acids were present in both *P. pastoris* and *S. cerevisiae* autolysates (Figure 6a). Remarkably, amino acid concentrations in *S. cerevisiae* autolysates, which performed poorly in the full basal medium substitution experiment, were substantially higher than in *P. pastoris* autolysate, which performed better (Figure 3). This is a strong indication that these differences in proliferation efficiencies were not caused by free amino acids.

We further analyzed waste medium components’ concentration after passage 3. The concentrations of the amino acids in the soy hydrolysate with *P. pastoris* autolysate waste medium were not diminished, but higher than in the fresh medium (Figure 6b). This may be caused by the quite substantial evaporation during the cultivation in six-well plates, but can also be the result of peptidase activity in spent media, hydrolyzing extracellular peptides, as was demonstrated in earlier studies [51].

To further test the importance of free amino acids in HBBM, the following amino acids were identified as possible bottlenecks for *P. pastoris* autolysate, due to much lower concentrations compared to DMEM/F12, based on the performed analysis (Figure 6a,b): Arg, His, Met, Trp (essential), Cys, and Gln (non-essential). These potentially critical amino acids were supplemented to the corresponding media, containing only *P. pastoris* autolysate as a basal media alternative. Still, upon supplementation, yeast autolysates performed much worse as compared to DMEM/F12 in short-term experiments (Figure S7), once again supporting the conclusion that amino acid composition and their concentrations are not the most critical bottleneck of the HBBM for BSCs in cell culture. 

### 3.8. Analysis of the Vitamin Content of the Hydrolysates

Next, vitamins in the yeast cell extracts, in yeast autolysates and in soy hydrolysate were analyzed and compared to DMEM/F12 concentrations (Table 1). From six essential vitamins (B_1_, B_2_, B_3_, B_5_, B_6_ and B_9_) two—B_2_, B_9_—were below the limit of detection in both *P. pastoris* autolysate and soy hydrolysate, used for the final HBBM (Table 1) [52]. The same was true for another two vitamins, which are not essential, but are included in DMEM/F12 formulation for better performance—B_7_ and B_12_.

### 3.9. Analysis of the Organic Compounds and Metal Ion Content of the Hydrolysates

The analysis of organic compounds detected in the hydrolysates revealed two chemicals that could potentially be toxic to mammalian cells—formic acid and ethanol (Table S1). However, the detected concentrations of both substances were significantly lower than levels reported to be toxic to mammalian cells. Further, ethanol was detected in both *S. cerevisiae* and *P. pastoris* autolysates, but not in soy hydrolysate or in the waste medium (Table S1).

Interestingly, low concentrations of Ala-Gln, which is used in cell culture as a more stable alternative to Gln, were detected in *S. cerevisiae* autolysate batch 2, and at a higher concentration in soy plant hydrolysate. This could be one of the reasons why yeast extracts performed better when supplemented with soy plant hydrolysate (Figure 3).

Contamination with heavy metals (arsenic, cadmium, mercury, lead, etc.) in these samples was not detected (Table S2), but caution should be taken when a new biomass source or extraction method is being introduced.

### 3.10. Life Cycle Analysis

**Figure 7:**
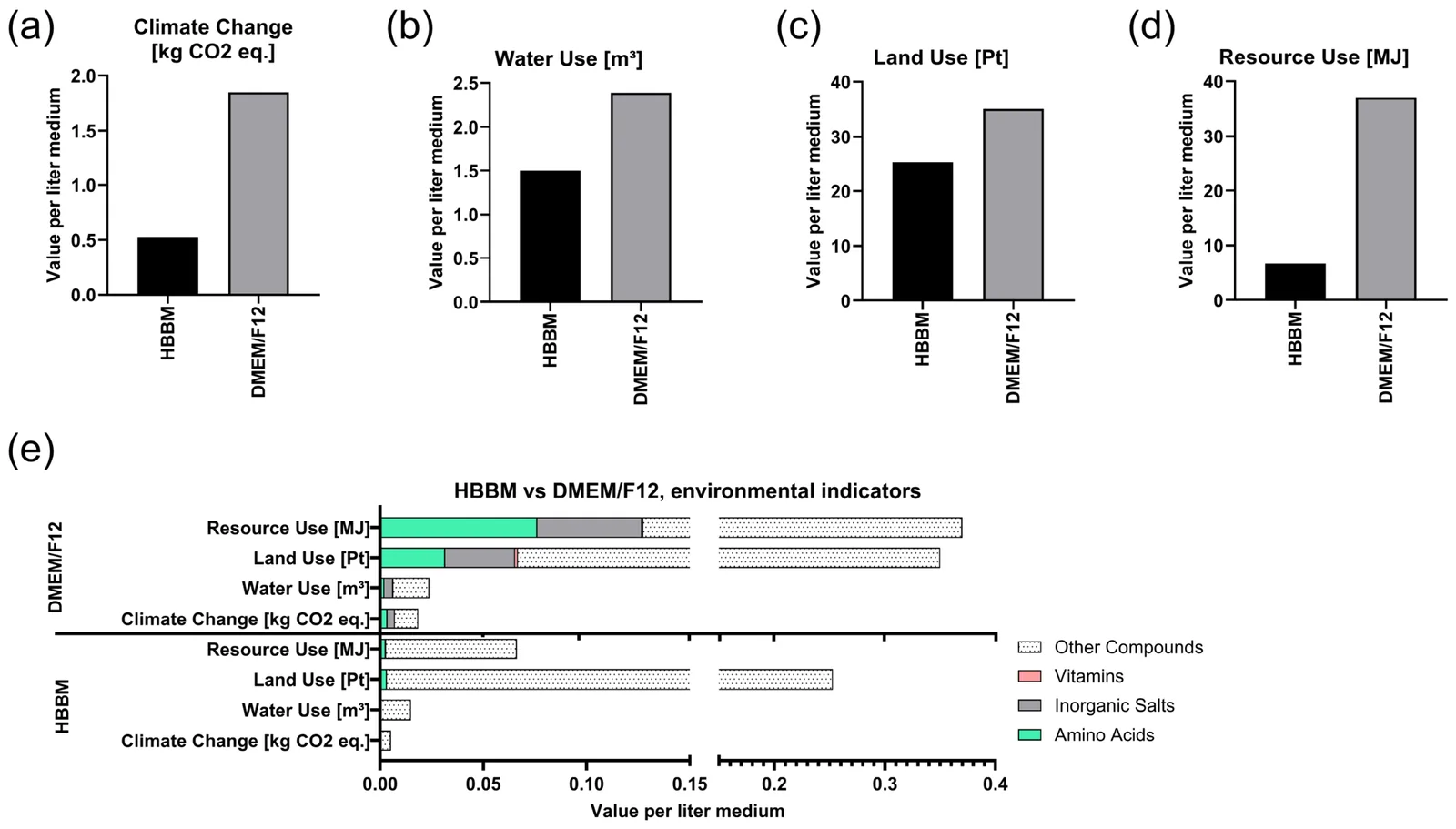
Life cycle analysis of the amino acids, vitamins and minerals in HBBM and DMEM/F12 medium. Comparison between HBBM and DMEM/F12 on four different environmental indicators—(**a**) climate change, (**b**) water use, (**c**) land use, and (**d**) resource use. (**e**) Contribution of different media components to the environmental impacts of basal medium—comparison between HBBM and DMEM/F12.

Furthermore, we examined whether the use of HBBM would offer an environmental benefit over DMEM/F12. Figure 7 presents the ecological impacts of HBBM and DMEM/F12 across four environmental indicators. A table with absolute values is given in the Supplementary Material (see Tables S3 and S4; used datasets are listed in Table S5). The LCA results indicate the environmental advantage of the HBBM relative to standard DMEM/F12 across all assessed categories. This is owed to a much lower contribution of inorganic salts and amino acids in HBBM, leaving “Other Compounds”, consisting mainly of glucose, as the main contributor (Figure 7e).

As a climate change/environmental impact indicator, HBBM emits ~3.5 times less CO_2_ equivalent per liter (Figure 7a), indicating a lower contribution to greenhouse gas emissions. Water use shows that HBBM utilizes ~1.6 times less water than DMEM/F12 (Figure 7b). HBBM would require approximately 1.4 times less land than DMEM/F12, whereas the most dramatic difference was found in the environmental indicator of resource use, where HBBM utilizes approximately 5.6 times less energy, suggesting much less energy-intensive raw material production or processing.

The lower climate change impacts observed for HBBM are associated with a reduced contribution from energy-intensive chemical synthesis and purification processes compared to the fully defined DMEM/F12 formulation. Similarly, lower water use for HBBM involves hydrolysis and basic filtration, which is a relatively water-lean processing method, while individual DMEM/F12 components go through separate purification and washing cascades, compounding the water burden.

Land use difference is the smallest among the discussed environmental indicators for HBBM, though hydrolysates from plant sources like soy or rice still have an agricultural footprint, as both media ultimately trace back to land. However, DMEM/F12’s amino acids go through an additional fermentation/synthesis step on top of crop-derived feedstocks, pushing its land score higher.

The resource use for HBBM includes production of bulk nutrient mixture in far fewer steps than the chemical synthesis of DMEM/F12. These differences reflect variation in upstream supply chains and processing requirements rather than changes in the functional performance of the media. Taken together, the results demonstrate that alternative basal media formulations can substantially influence environmental performance, with HBBM reducing the ecological burden. These findings provide a quantitative basis for assessing sustainability implications under future scale-up scenarios.

### 3.11. Limitations of the Study

The primary limitation affecting the sustainability of plant and yeast hydrolysates used in the current study is the lack of carbon sources in these hydrolysates. Both yeast and plant hydrolysates contained no glucose or alternative metabolites that can be readily utilized by mammalian stem cells. However, after FBS and amino acids, glucose is the third most significant component of the cell culture medium contributing to environmental impacts (see [8] and Figure 7e). Since most glucose is produced from starch derived from grains, such as corn and wheat, it has a considerable impact on land use [8]. Therefore, exploring alternative sources of glucose is recommended. For example, microalgae could provide glucose with a markedly smaller environmental footprint compared to glucose obtained from grains [53]. Additionally, microalgae demonstrate CO_2_ fixation rates that are 10 to 50 times higher than those of terrestrial plants, owing to the presence of CO_2_ concentration mechanisms [54], which could enhance the feasibility of such applications.

Additionally, the big challenge associated with hydrolysate utilization—batch-to-batch variability—should be examined in greater detail than done in the present study, including long-term experiments. Achieving greater consistency between batches would offer significant advantages for process monitoring. Furthermore, it remains unclear how significantly variations in free amino acid composition, demonstrated in Figure S6, affect cell proliferation, considering that mammalian cells can also utilize short peptides [13,14,15], which were not monitored in this study due to the limitation of the analytical methods used—the BCA assay can only detect peptides and proteins with more than 18 amino acids or 2000 Da [55].

Furthermore, this study did not conclude safety research, necessary prior to application of such hydrolysates in the food industry. Their precise characterization must be carried out, including defined release specifications, residual enzyme and allergen profiles, sterilization compatibility, storage conditions and shelf-life.

Next, in the LCA, the reported percentage change is subject to uncertainties associated with inventory data quality and modeling assumptions. Life cycle inventories for culture medium components are based on secondary data and may not fully reflect industrial-scale production efficiencies. Additionally, system boundaries exclude certain downstream processes, such as spent media treatment, recycling, and waste management, which may influence absolute impact levels. Variations in energy mixes, production technologies, and supplier-specific practices further contribute to uncertainty.

## 4. Discussion

We conclude that DMEM/F12 and an alternative, hydrolysate-based basal medium (HBBM) (e.g., consisting of 0.1 g/L soy hydrolysate combined with 0.025 g/L *P. pastoris* autolysate, supplemented with glucose and additionally stabilized by 0.1 g/L methyl cellulose) perform comparably in supporting the proliferation and differentiation of BSCs in short- and long-term experiments. As we discuss below, first assessments show that HBBM could be significantly more economical and environmentally friendly than DMEM/F12. However, before employing such a basal medium substitute in food industry, the technology must be further optimized, thoroughly tested for safety, and scaled up to a pilot level, incorporating a comprehensive techno-economic assessment.

Medium costs are a central focus of optimization in cultured meat production, and HBBM could represent a new perspective for such optimization. The market price of plant protein hydrolysates ranges from €1.80 to €28.00 per kg [25], though NaTurtle has internally assessed the costs for the scaled production of its soy hydrolysates at 40 €/kg, which we applied for the calculation below. For the production of *P. pastoris* autolysate, the same method and facilities can be used as are currently used for production of baker’s yeast extract, with an estimated price equal to the *S. cerevisiae* yeast extract of 40 €/kg. Keeping in mind the limitations of the current laboratory-scale study, an order-of-magnitude projection of HBBM price can be performed. With the given concentrations (0.1 g/L soy hydrolysate combined with 0.025 g/L *P. pastoris* autolysate and 0.1 g/L methyl cellulose), the price contribution for the HBBM is estimated at around 7 cent/L, whereas standard DMEM/F12’s price is around 2.5 €/L bulk (€125.00/50 liter in powder form (Gibco™ DMEM/F-12 #32500043; Fisher Scientific #11504466)). This result predicts an approximate 36-fold reduction in the price of the basal medium, although a techno-economic assessment would be required to estimate it more realistically.

The comparative LCA shows that basal culture medium formulation plays a key role in shaping the environmental performance of cultured meat systems. The results suggest that replacing chemically fully defined media with alternative formulations can significantly lower emissions. This supports earlier findings that chemical synthesis and purification of single media components are major climate hotspots. From a scale-up perspective, these findings indicate that medium innovation represents a critical sustainability lever, potentially outweighing improvements at the bioprocess or facility level. Future work should integrate primary industrial data, assess sensitivity to energy scenarios, and expand system boundaries to better quantify the long-term environmental impacts of alternative culture media at a commercial scale.

Additionally, to facilitate efficient upscaling, further research is necessary to elucidate the mechanisms and potential limitations of HBBM, as several of the following important questions remain unanswered.

The efficiency of *P. pastoris* autolysis was quite low and, as a result, the best working combination of HBBM contained much lower amino acid concentrations than used in DMEM/F12 composition (Figure 6a). If free amino acids are more preferably consumed as compared to peptides, their lower concentrations could lead to fast depletion of high-demand amino acids in high-cell-density cultures. On the other hand, as hydrolysis was not exhaustive, a substantial portion of amino acids would be present in the form of peptides, which are efficiently cleaved by proteases in cell culture or directly taken up by the mammalian cells [13,14,15]. Previous studies have also demonstrated that peptides could be better absorbed than some of the free amino acids [56,57]; they have a higher solubility and stability, and they can have significant positive effects on cells’ metabolism in culture, although the mechanisms are not fully understood [51,58]. These advantages of peptides over free amino acids might explain the better performance of *P. pastors* hydrolysate despite lower free amino acid concentrations (Figure 4 and Figure 6a), as well as the fact that waste HBBM demonstrated slightly higher free amino acid concentrations than the fresh HBBM (Figure 6b). However, more research must be done to validate these assumptions.

Furthermore, although soy hydrolysates had consistently demonstrated a better performance in long-term experiments than wheat and rice hydrolysates, the reason for that stays unclear. Wheat hydrolysates were very efficient in promoting proliferation in serum-free formulations for CHO [15,59] and even for a fish cell line [60], although not as a substitute of the basal medium, but additionally to it. In this setup, for CHO cells, a mixture of 60% of soy hydrolysate and 40% of wheat gluten hydrolysate led to the highest maximum viable cell concentration, whereas a mixture of soy and wheat hydrolysate with yeastolate led to a higher recombinant antibody production [59]. Also, when rice and wheat protein hydrolysates’ addition to basal protein-free CHO cell culture medium was compared, wheat protein hydrolysates demonstrated a higher CHO cell density [15]. However, to our knowledge, no studies have been performed so far where a full substitution of basal medium with plant hydrolysates has been attempted. In this setup, an absence of any critical cell culture component—essential amino acids, vitamins or mineral ions—is devastating. Additionally, as in the case of yeast autolysates, the degree of hydrolysis, type of enzyme, pH, separation and filtration steps, and drying procedure could have a great impact on the hydrolysate content [2]. We conclude that more research has to be done to determine the reasons why wheat or rice hydrolysates were underperforming in long-term experiments, or why soy hydrolysate had a significant advantage.

Meanwhile, we can conclude with a reasonable degree of certainty that lower amino acid concentrations in the alternative basal medium and depletion during cultivation are not among the most critical bottlenecks of HBBM.

Further, the analysis has shown that two (B_2_ and B_9_) out of six essential vitamins (B_1_, B_2_, B_3_, B_5_, B_6_ and B_9_) were below the limit of detection in the *P. pastoris* autolysate and soy hydrolysate, used for the final HBBM (Table 1). B_2_ (riboflavin) is an antioxidant, a coenzyme in carbohydrate and lipid metabolism, which is also involved in activation of vitamins B_6_ and B_9_, and is naturally produced by plants [61] and *P. pastoris* [62]. Its normal concentration in blood ranges from 174 to 471 nM, which is somewhat below the detection level of the analytical methods used in this study, 0.156 μM, as well as below the concentration used in DMEM/F12—0.5824 μM. Its lower concentration in blood suggests that the required concentrations are below those used in DMEM/F12, and could explain the fact that BSCs were still able to proliferate and differentiate in HBBM.

The same holds true for B_9_ (folic acid), which is an essential vitamin and a co-enzyme involved in one-carbon metabolism and in epigenetic and direct regulation of nucleotide synthesis and amino acid metabolism. Vitamin B_9_ is naturally produced by plants and yeasts in various forms, collectively called folates [63], and its normal concentration in blood is >3.0 nM [52], which is significantly below that used in DMEM/F12 (6.009 μM) and below the limit of detection of the analytical methods used in this study (0.03 μM).

Additionally, B_7_ (biotin) and B_12_ (cobalamin), which are often used in basal medium, were below the limit of detection. Here, the same explanation as above is suggested.

We conclude that low concentrations of multiple vitamins could result in another bottleneck of HBBM during the scale-up, as they are present in DMEM/F12 at higher concentrations (Table 1). Further optimization of methods for the preparation of hydrolysates should be undertaken, to minimize the loss of vitamins.

Also, although no heavy metals were detected in the HBBM, other questionable substances were detected at non-alarming concentrations.

Ethanol was detected in both yeast autolysates (HBBM concentration 0.1802 mM). Ethanol is traditionally used as a preservative in food at concentrations of 0.5% to 70%, whereas lower concentrations (1–2 mM and 0.1%) were reported to display a cytoprotective effect in certain cell types, e.g., hepatocytes and hippocampal cells [64,65].

For formic acid, that damages cells by inhibiting mitochondrial respiration and causing oxidative stress, concentrations higher than 25 mM lead to chromosomal aberrations in CHO K1 cells, and 106 mM (0.2%) leads to slight cytotoxicity in Jurkat and NIH 3T3 cells [66,67]. On the other hand, low concentrations of formic acid (0.01–0.02% or 5.3–10.6 mM) are used as a preservation agent in the food industry (E 236) to inhibit bacterial and fungal growth, extending shelf life in products like pickled foods, juices, and some meats. The concentrations detected in soy plant hydrolysate and in the waste medium were in the range of 0.0078 to 0.0493 mM (Table S1), which is about 100 times lower than necessary for conservation purposes. The formic acid concentration was approx. six times higher in waste medium than in fresh HBBM—further studies should elucidate its potential for accumulation, which could eventually lead to cytotoxic concentrations.

We conclude that while HBBM offers a highly promising approach to reducing basal medium costs and enhancing sustainability, additional research and validation are necessary to ensure its safety and consistency before it can be considered as a substitute for chemically fully defined basal medium in cultured meat production.

## Figures and Tables

**Table 1:** Vitamin contents of the yeast cell extract, yeast autolysate and soy hydrolysate compared to DMEM/F12. Analyzed concentrations were adapted to reflect the best working concentrations in the cell culture media (0.1 g/L soy hydrolysate combined with 0.025 g/L *P.p.* autolysate). *P.p.*, *Pichia pastoris*; *S.c.*, *Saccharomyces cerevisiae*; <LOD, below limit of detection.

Vitamins, µM	*S.c.* Cell Extract Batch 2	*S.c.* Autolysate Batch 2	*S.c.* Cell Extract Batch 3	*S.c.* Autolysate Batch 3	*P.p.* Cell Extract Batch 1	*P.p.* Autolysate Batch 1	Soy Plant Hydrolysate	HBBM	DMEM/F-12, HEPES, µM
4-Aminobenzoic acid	<LOD	0.0006	<LOD	<LOD	0.0008	<LOD	0.0018	0.0018	0
Biotin (B_7_)	<LOD	<LOD	<LOD	<LOD	<LOD	<LOD	<LOD	<LOD	0.0143
Choline	0.0529	0.6716	0.3449	0.3756	0.0086	0.0480	0.3549	0.4030	64.1429
Cyanocobalamin (B_12_)	<LOD	<LOD	<LOD	<LOD	<LOD	<LOD	<LOD	<LOD	4.6960
Folic acid (B_9_)	<LOD	<LOD	<LOD	<LOD	<LOD	<LOD	<LOD	<LOD	6.0091
Niacin (B_3_)	0.2334	0.2420	0.2780	0.2938	0.0038	0.2343	0.0084	0.2427	16.5574
Nicotinamide (B_3_)	<LOD	0.4115	<LOD	<LOD	0.0470	0.0318	0.0110	0.0428	9.7087
Panthotenic acid (B_5_)	0.0032	0.0453	0.0174	0.0198	0.0038	0.0215	0.0010	0.0225	0.5824
Pyridoxal (B_6_)	<LOD	0.0004	0.0012	0.0009	0.0002	<LOD	<LOD	<LOD	6.4392
Pyridoxamine (B_6_)	0.0008	0.0205	0.0112	0.0119	0.0007	0.0033	<LOD	0.0033	0.5018
Pyridoxine (B_6_)	0.0029	0.0009	0.0027	0.0031	<LOD	<LOD	<LOD	<LOD	70.0000
Riboflavin (B_2_)	<LOD	0.0132	<LOD	<LOD	0.0016	<LOD	<LOD	<LOD	0.5824
Thiamine (B_1_)	0.0024	0.0676	0.0207	0.0234	0.0032	0.0149	0.0020	0.0170	6.4392

## Data Availability

The original contributions presented in this study are included in the article/Supplementary Material. Further inquiries can be directed to the corresponding authors.

## Supplementary Materials

The following supporting information can be downloaded at https://www.mdpi.com/article/10.3390/foods15183207/s1: Figure S1: Production of the yeast autolysates from two yeast species. Figure S2: Short-term toxicity tests of the produced lysates in the serum-free medium B9 + MC, using BSCs as reporter cells. Figure S3: BSC proliferation in a full substitution of DMEM/F12 basal medium with yeast hydrolysates. Figure S4: Differentiated BSCs, cultured in the best performing combination containing *P.p.* hydrolysate and in the best performing combination of *S.c.* hydrolysate. Figure S5: Morphology of the non-differentiated BSCs on day 4 upon full basal medium substitution with HBBM. Figure S6: Comparison of two *S.c.* autolysates to assess batch-to-batch variations. Figure S7: BSC proliferation in a full substitution of DMEM/F12 basal medium with yeast hydrolysates supplemented with a mix of amino acids. Table S1: Organic compounds detected in the yeast cell extract, yeast autolysate, soy hydrolysate, and waste media (B8 + MC with 0.1 g/L soy hydrolysate combined with 0.025 g/L *P.p.* autolysate as a basal medium), compared to DMEM/F12. Table S2: Elements detected in the hydrolysate samples, compared to DMEM/F12. Table S3: Ecological impacts of the composition of HBBM and DMEM/F12 on different environmental indicators. Table S4: Percentage ratios of the ecological impacts of HBBM and DMEM/F12 components from Table S3 on different environmental indicators. Table S5: The datasets used for the LCA analysis.

## Abbreviations

The following abbreviations are used in this manuscript:

DMEM/F12	1:1 Dulbecco’s Modified Eagle Medium/Nutrient Mixture F-12
B8	HiDef-B8 medium
B9	HiDef-B8 medium supplemented with 0.8 g/L HSA
BHK	Baby Hamster Kidney cells
BSCs	Bovine Satellite Cells
CHO	Chinese Hamster Ovary cells
CM	Cultured Meat
CWW	Cell Wet Weight
ddH_2_O	Double Distilled Water
DMSO	Dimethyl Sulfoxide
DPBS	Dulbecco′s Phosphate-Buffered Saline
EDTA	Ethylenediaminetetraacetic acid
EFSA	European Food Safety Authority
FBS	Fetal Bovine Serum
FDA	US Food and Drug Administration
FGF-2	Fibroblast growth factor 2
GM	Growth Medium
Ham’s F10	Ham’s F-10 Nutrient Mix
HBBM	Hydrolysate-Based Basal Medium
HEK	Human Embryonic Kidney cells
HSA	Human Serum Albumin
LCA	Life Cycle Assessments
LC-MS/MS	Liquid Chromatography–Tandem Mass Spectrometry
MC	Methyl Cellulose
NMR	Nuclear Magnetic Resonance
*S.c.*	*Saccharomyces cerevisiae*
*S. cerevisiae*	*Saccharomyces cerevisiae*
*P. pastoris*	*Pichia pastoris*
*P.p.*	*Pichia pastoris*
RSD	Relative Standard Deviation
VERO	Kidney epithelial cells isolated from the African green monkey
YPD	Yeast–Peptone–Dextrose broth
