# Supplementary material for: Highly Sustainable Plant and Yeast Hydrolysate-Based Alternative for Basal Medium Supports Long-Term In Vitro Propagation of Bovine Satellite Cells
Source: Foods. 2026 Sep 10;15(18):3207. doi: 10.3390/foods15183207 (PMC13605773; doi:10.3390/foods15183207)
Supplement: Supplementary file 1 [file foods-15-03207-s001.zip › foods-4487564-supplementary.pdf]

Supplementary Materials

Article

Highly Sustainable Plant and Yeast Hydrolysate-Based Alternative for Basal Medium Supports Long-Term *In Vitro* Propagation of Bovine Satellite Cells

Lisa Schenzle [1], Shashank Goyal [2], Regina Leber [1], Harald Pichler [1,3,*] and Aleksandra Fuchs [1,4,*]

[1] acib—Austrian Centre of Industrial Biotechnology, Graz 8010, Austria; lisa.schenzle@medunigraz.at (L.S.)

[2] EurA AG, Erfurt 99084, Germany; shashank.goyal@eura-ag.de

[3] NAWI Graz, BioTechMed Graz, Institute of Molecular Biotechnology, Graz University of Technology, Graz 8010, Austria

[4] Division of Physiology, Otto Loewi Research Center for Vascular Biology, Immunology and Inflammation, Medical University of Graz, Graz 8010, Austria

* Correspondence: al.fuchs@medunigraz.at (A.F.); harald.pichler@tugraz.at (H.P.)

(a) Yeast cell autolysates

	Species	Protein content after hydrolysis
1.	*S. cerevisiae* batch 1	11.42 g/L
2.	*S. cerevisiae* batch 2	7.2 g/L
3.	*P. pastoris* batch 1	0.8 g/L

(b)

Abbreviations:
S.c. – *Saccharomyces cerevisiae*
P.p. – *Pichia pastoris*
YCE – yeast cell extract
AL – yeast autolysate
B1/B2 – Batch 1 or batch 2
10 µg/20 µg – Total protein quantity loaded, as determined by BCA

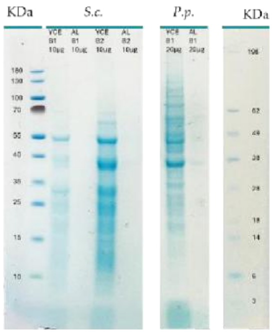

Figure S1: Production of the yeast autolysates from two yeast species. **(a)** List of the species and batches produced to compare the inter-species yields and batch-to-batch variations. **(b)** A high degree of autolysis was achieved, as demonstrated via comparison of the digested and non-digested samples on the SDS-PAGE.

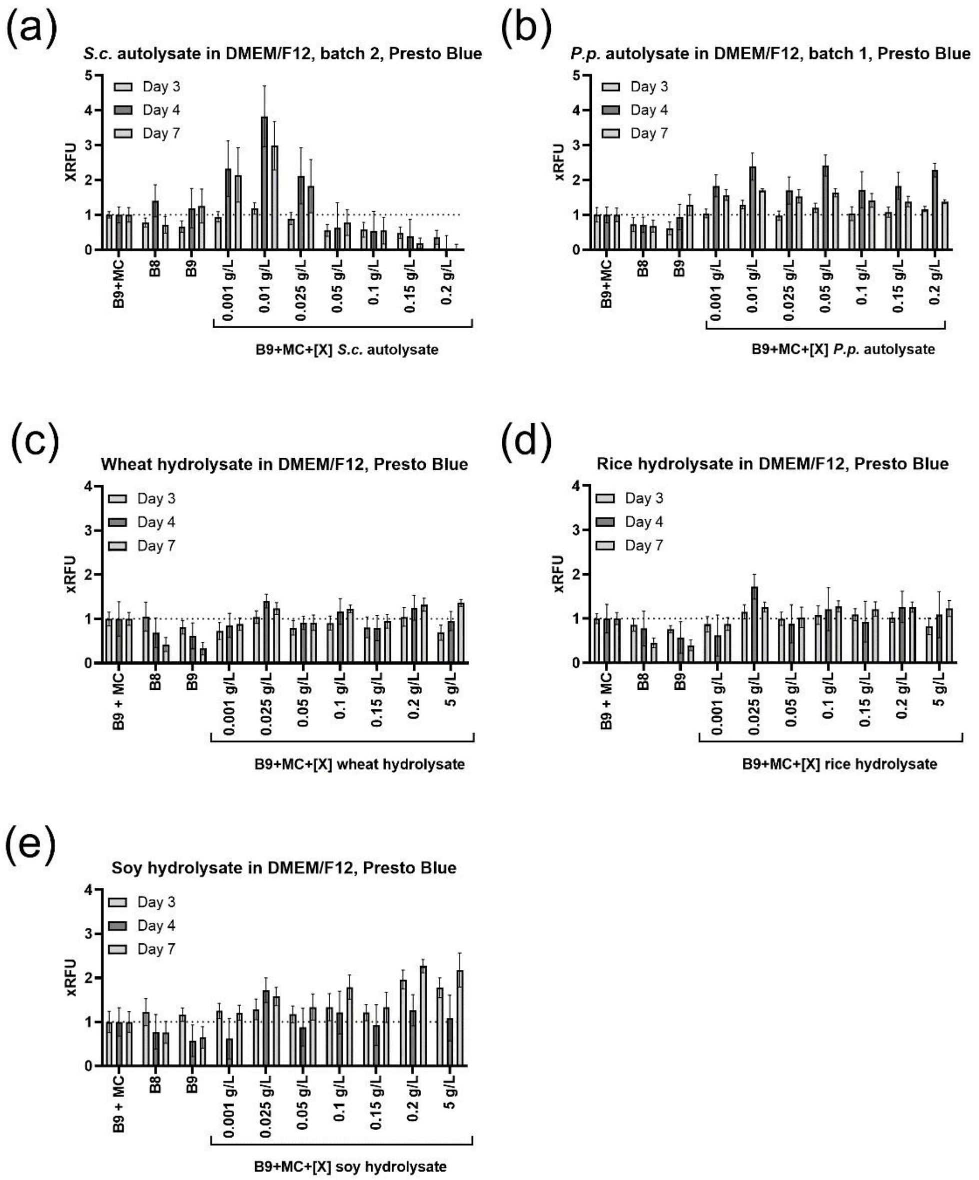

Figure S2: Short-term toxicity tests of the produced lysates in the serum-free medium B9+MC, using BSCs as reporter cells. 2000 BSCs/cm2 were seeded on day 0 in GM and changed on day 1 to the designated medium. (a-b) Yeast autolysates and (c-e) plant hydrolysates in a range of concentrations were supplemented to the B9+MC medium and, as indicated, Presto Blue assay (days 3,4,7) was performed to assess the viability and proliferation rate of the BSCs. Obtained values were normalized to BSCs in B9+MC. n=6 and repeated twice. *P.p., Pichia pastoris*; *S.c. Saccharomyces cerevisiae*; MC, methyl cellulose.

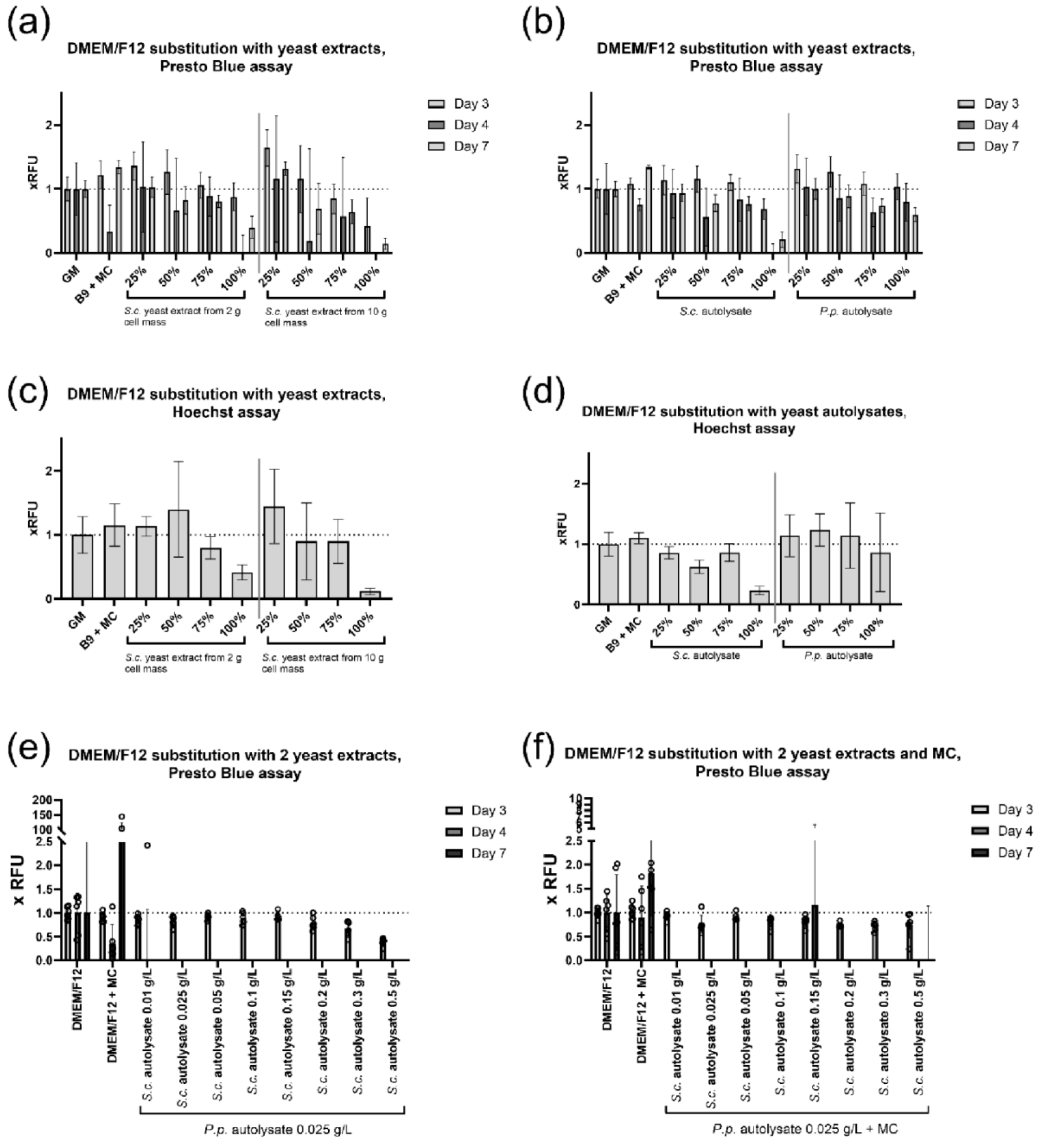

Figure S3: BSCs proliferation in a full substitution of DMEM/F12 basal medium with yeast hydrolysates. Presto Blue and Hoechst assay of the BSCs proliferation in a successive substitution of DMEM/F12 with **(a-b)** *S. cerevisiae* yeast extracts (not hydrolysed) or **(c-d)** *S. cerevisiae* or *P. pastoris* autolysates in growth medium (20% FBS + 1 ng/mL FGF-2). The percentage on the x-axis reflects the concentration of yeast-based basal medium, e.g. with 25% being 75% of DMEM/F12 + 25% of yeast extract. **(e-f)** Both *S. cerevisiae* and *P. pastoris* yeast autolysates were combined in given concentrations and tested as a full substitution of DMEM/F12 in serum free B9, supplemented with glucose to DMEM levels. *P.p., Pichia pastoris; S.c. Saccharomyces cerevisiae;* MC, methyl cellulose.

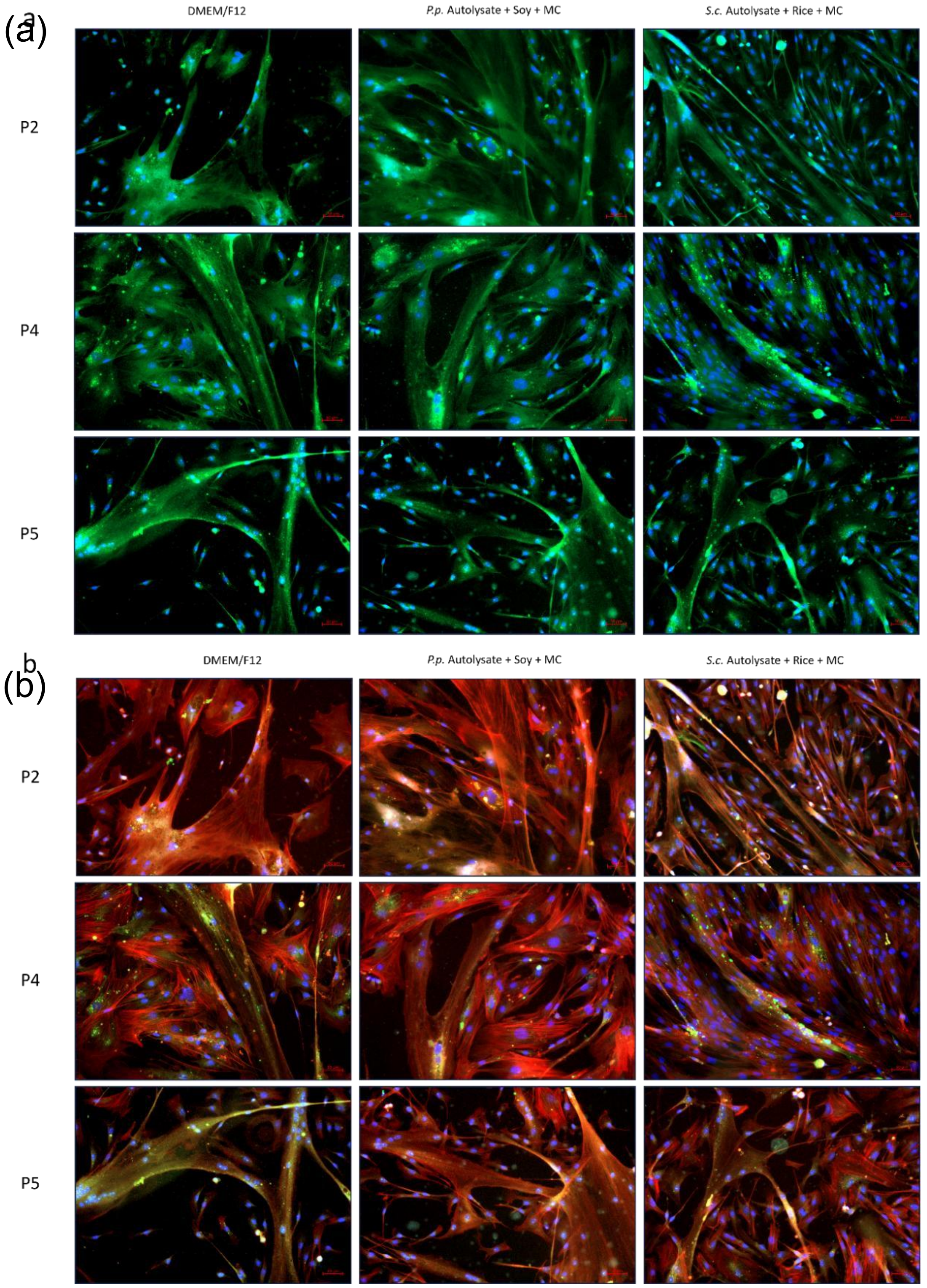

Figure S4: Differentiated BSCs, cultured in the best performing combination containing *P.p.* hydrolysate and in the best performing combination of *S.c.* hydrolysate. BSCs cells were cultured in 6-well plates in B9 add-on media, using as basal medium either DMEM/F12 or a combination of 0.1 g/L plant hydrolysates combined with 0.025 g/L yeast autolysates named HBBM. Both hydrolysates were added immediately upon splitting, whereas HSA of the B9 add-on medium was added 24h after the splitting. Upon each passage, part of the cells was seeded for differentiation and IF staining. Immunofluorescence staining was performed for nuclei (DAPI, blue), and for muscle differentiation markers **(a)** desmin (green) and **(b)** additionally phalloidin (red). Scale bar = 50 µm. Microscope Zeis Axio Imager. Lens: EC Plan-Neofluar 10x; Exposure DAPI: 20 ms. *P.p., Pichia pastoris; S.c. Saccharomyces cerevisiae;* MC, methyl cellulose.

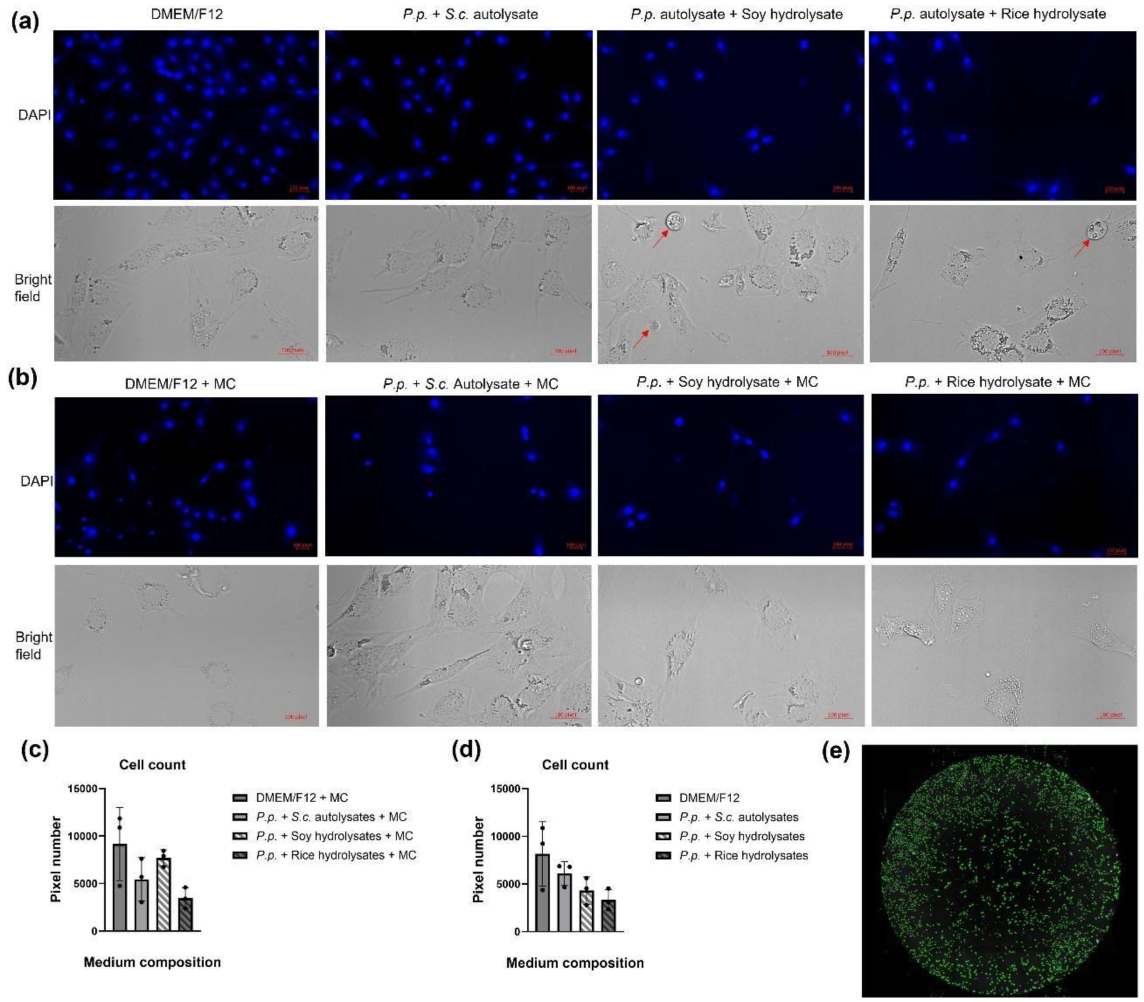

Figure S5: Morphology of the non-differentiated BSCs on day 4 upon full basal medium substitution with HBBM. BSCs cells were cultured in 96-well glass bottom plates (Thermo Scientific #165305) in B9 add-on media, using as basal medium either DMEM/F12 or a combination of 0.1 g/L soy hydrolysate combined with 0.025 g/L *P. p.* autolysate. HSA (as a part of B9 add-on medium) was added 24h after the splitting. For assessing cell count, cells were cultivated in designated media **(a)** without or **(b)** with MC, and on day 4 nuclei were stained with DAPI (blue). For evaluating possible morphological changes, Bright field images were inspected. Red arrows indicate detached cells. DAPI images are zoomed in 67%, Bright field images are zoomed in 100%. Image acquisition (Bright field and DAPI) was performed in collaboration with Medical University of Graz (Group Brislinger) using Olympus IX83 inverted microscope (Evident Scientific) with a 20x phase contrast objective (Na=0.7, UCPLFLN20XPH), using Olympus cellSens Dimension software (Version 4.1.1.) with the WellNavigator-tool; VIVO-HP-B-3 Incubation-housing with temperature and CO_2 control. Images taken with CMOS-Camera HAMAMATSU Orca Flash 4.0. Scale bar = 100 µm. **(c-d)** DAPI cell count was performed by the Zeiss Zen cell counting tool using 3 whole wells per condition. **(e)** Example of the full well DAPI stained cells. *P.p.*, *Pichia pastoris; S.c., Saccharomyces cerevisiae;* MC, methyl cellulose.

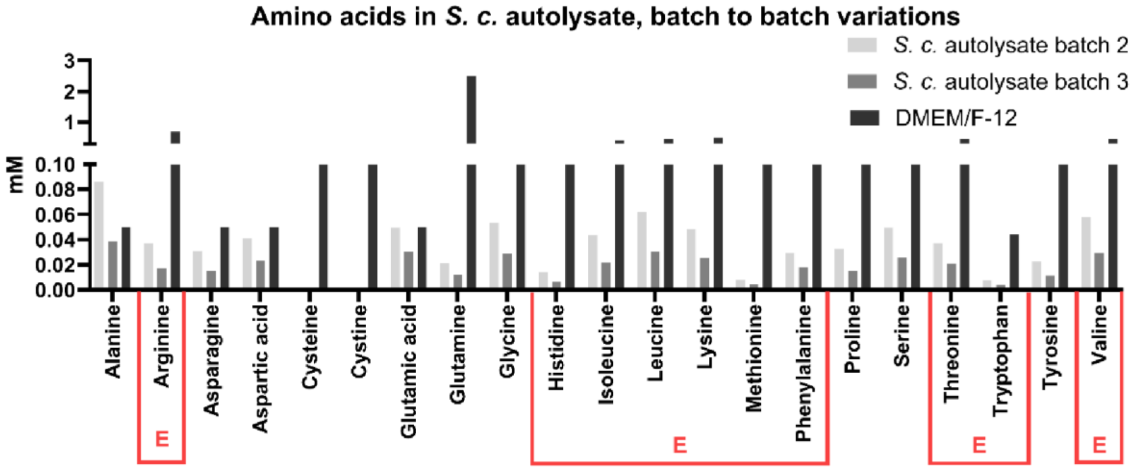

Figure S6: Comparison of two *S. c.* autolysates to assess batch-to-batch variations. DMEM/F12 contents are plotted as a control. Analysed concentrations were adapted to reflect the best working concentrations in the cell culture media (0.1 g/L soy hydrolysate combined with 0.025 g/L *P. p.* autolysate).

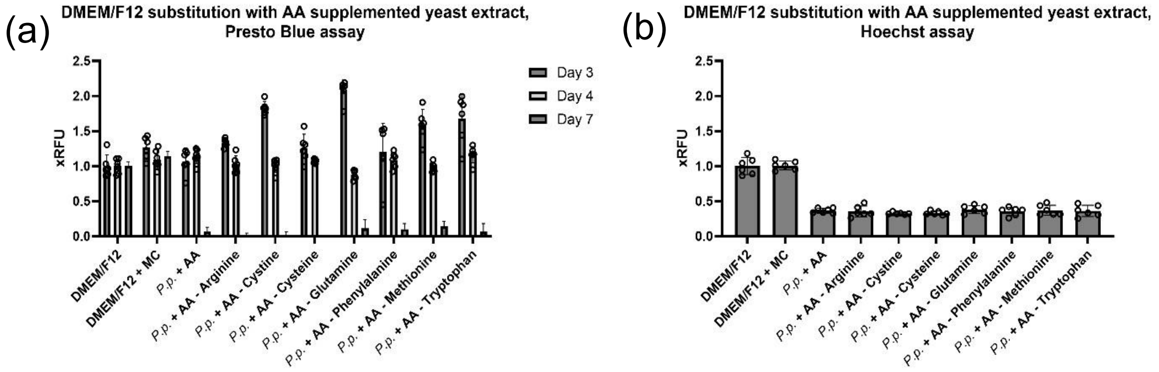

Figure S7: BSCs proliferation in a full substitution of DMEM/F12 basal medium with yeast hydrolysates supplemented with a mix of amino acids. **(a)** Presto Blue and **(b)** Hoechst assay of the BSCs proliferation in serum free B9 medium (+MC) with either DMEM/F12 as basal medium (controls) or with *P. pastoris* hydrolysates as basal medium. In *P.p.* + AA, *P. pastoris* hydrolysates were supplemented with all critical amino acids (Arg, His, Met, Trp (Essential) and Cys, Gln (Non-essential)) at concentrations present in DMEM/F12. E.g. in *P.p.* + AA - Arginine, all critical amino acids were present, except of Arginine. MC, methyl cellulose.

Organic compounds, mM	*S. c.* cell extract Batch 2	*S. c.* autolysate Batch 2	*S. c.* cell extract Batch 3	*S. c.* autolysate Batch 3	*P. p.* cell extract Batch 1	*P. p.* autolysate Batch 1	Waste cell culture media	Soy plant hydrolysate	HBBM	DMEM/F-12, HEPES
**Acetic acid**	0.0050	0.0292	0.0061	0.0067	0.0075	0.1343	0.1124	0.0329	0.1672	0
**Butyric & 2-hydroxy-butyric acids**	< LOD	< LOD	< LOD	< LOD	< LOD	< LOD	< LOD	< LOD	< LOD	0
**3-Hydroxybutyric acid**	< LOD	< LOD	< LOD	< LOD	< LOD	< LOD	< LOD	< LOD	< LOD	0
**Citric acid**	< LOD	< LOD	< LOD	< LOD	< LOD	< LOD	< LOD	0.0950	0.0950	0
**Formic acid**	< LOD	< LOD	< LOD	< LOD	< LOD	< LOD	**0.0493**	**0.0078**	**0.0078**	0
**Fumaric acid**	< LOD	< LOD	< LOD	< LOD	< LOD	< LOD	< LOD	< LOD	< LOD	0
**Isovaleric acid**	< LOD	< LOD	< LOD	< LOD	< LOD	< LOD	< LOD	< LOD	< LOD	0
**Lactic acid**	< LOD	< LOD	< LOD	< LOD	< LOD	< LOD	0.1707	< LOD	< LOD	0
**Maleic acid**	< LOD	< LOD	< LOD	< LOD	< LOD	< LOD	< LOD	< LOD	< LOD	0
**Propionic acid**	< LOD	< LOD	< LOD	< LOD	< LOD	< LOD	< LOD	< LOD	< LOD	0
**Pyruvic acid**	0.0074	< LOD	< LOD	< LOD	< LOD	< LOD	< LOD	< LOD	< LOD	0.5
**Succinic acid**	< LOD	0.0078	< LOD	0.0033	< LOD	0.0024	< LOD	0.0030	0.0054	0
**Acetone**	< LOD	< LOD	< LOD	< LOD	0.0020	< LOD	< LOD	< LOD	< LOD	0

**Ethanol**	**0.1172**	**0.0590**	**0.4370**	**0.0141**	**0.0936**	**0.1802**	< LOD	< LOD	**0.1802**	0
**Pyroglutamic acid**	< LOD	< LOD	< LOD	< LOD	< LOD	< LOD	< LOD	< LOD	< LOD	0
**Alanyl-glutamine**	< LOD	0.0001	< LOD	< LOD	< LOD	< LOD	< LOD	0.0059	0.0059	0

Table S1: Organic compounds detected in the yeast cell extract, yeast autolysate, soy hydrolysate, and waste media (B8 + MC with 0.1 g/L soy hydrolysate combined with 0.025 g/L *P. p.* autolysate as a basal medium, passage 3), compared to DMEM/F12. Analysed concentrations were adapted to reflect the best working concentrations in the HBBM containing cell culture media (0.1 g/L soy hydrolysate combined with 0.025 g/L *P. p.* autolysate). Underlined is pyruvic acid, present in DMEM/F12 but missing in most hydrolysates, **bold marked** are the potentially toxic compounds. *P.p., Pichia pastoris*; *S.c. Saccharomyces cerevisiae*; < LOD - below limit of detection.

mg/L	*S. c.* cell extract, batch 2	*S. c.* autolysate, batch 2	*S. c.* cell extract, batch 3	*P.p.* autolysate batch1	Soy plant hydrolysate	HBBM	DMEM/F-12, HEPES
**Ag**	< LOD	< LOD	< LOD	< LOD	< LOD	< LOD	
**Al**	< LOD	< LOD	< LOD	< LOD	< LOD	< LOD	
**As**	< LOD	< LOD	< LOD	< LOD	< LOD	< LOD	
**B**	< LOD	0.001707815	< LOD	0.001063772	< LOD	0.0011	
**Ba**	0.000514737	7.63494E-05	0.00127627	< LOD	0.000482335	0.0005	
**Be**	< LOD	< LOD	< LOD	< LOD	< LOD	< LOD	
**Ca**	0.094840277	0.149291156	0.126223092	0.014234289	0.242132308	0.2564	42.018016
**Cd**	< LOD	< LOD	< LOD	< LOD	< LOD	< LOD	
**Co**	< LOD	< LOD	< LOD	< LOD	< LOD	< LOD	
**Cr**	< LOD	< LOD	< LOD	< LOD	< LOD	< LOD	
**Cu**	< LOD	0.002684283	< LOD	< LOD	< LOD	< LOD	0.0003328
**Fe**	< LOD	0.00022503	< LOD	0.001006785	0.035210475	0.0362	0.090930693
**Hg**	< LOD	< LOD	< LOD	< LOD	< LOD	< LOD	
**K**	1.153396749	4.288017856	1.344805582	0.680722986	0.919083626	1.5998	162.1360026
**Li**	< LOD	< LOD	< LOD	< LOD	< LOD	< LOD	
**Mg**	0.264832144	0.721204439	0.237003318	0.047762118	0.194220337	0.2420	16.96791984
**Mn**	0.000643421	0.001048799	0.000893389	5.69878E-05	0.004180239	0.0042	
**Mo**	< LOD	< LOD	< LOD	< LOD	< LOD	< LOD	
**Na**	1.193623962	3.063390073	1.530673004	0.020407969	11.91159116	11.9320	3136.065699
**Ni**	< LOD	< LOD	< LOD	< LOD	< LOD	< LOD	
**P**	1.199337019	4.262093817	1.206202672	0.463285581	1.290729194	1.7540	
**Pb**	< LOD	< LOD	< LOD	< LOD	< LOD	< LOD	
**Rb**	< LOD	0.001418491	< LOD	< LOD	< LOD	< LOD	
**S**	0.164715814	0.678846401	0.272866502	0.093542327	0.78701039	0.8806	
**Se**	< LOD	< LOD	< LOD	< LOD	< LOD	< LOD	
**Si**	0.030626847	0.021112611	0.048370629	0.002855723	0.05820179	0.0611	
**Sn**	< LOD	< LOD	< LOD	< LOD	< LOD	< LOD	
**Sr**	0.000257368	0.00059874	0.000382881	4.43239E-05	0.000482335	0.0005	
**Ti**	< LOD	< LOD	< LOD	< LOD	< LOD	< LOD	
**Tl**	< LOD	< LOD	< LOD	< LOD	< LOD	< LOD	
**V**	< LOD	< LOD	< LOD	< LOD	< LOD	< LOD	
**Zn**	0.018659213	0.016672292	0.016336255	0.007300772	0.0081997	0.0155	0.0975

Table S2: Elements detected in the hydrolysate samples, compared to DMEM/F12. Analysed concentrations were adapted to reflect the best working concentrations in the HBBM containing cell culture media (0.1 g/L soy hydrolysate combined with 0.025 g/L *P. p.* autolysate). Red marked are heavy metals. *P.p., Pichia pastoris; S.c. Saccharomyces cerevisiae; < LOD - below limit of detection.*

**Environmental indicator**	**HBBM (value per liter)**	**DMEM/F12 (value per liter)**

Climate Change [kg CO_2 eq.]	5.28E-03	1.85E-02
Water Use [m³]	1.50E-02	2.39E-02
Land Use [Pt]	2.53E-01	3.50E-01
Resource Use [MJ]	6.63E-02	3.70E-01

Table S3: Ecological impacts of the composition of HBBM and DMEM/F12 on different environmental indicators.

Environmental indicator	Amino Acids	Inorganic Salts	Vitamins	Other Compounds
**DMEM/F12**				
Climate Change	19.4 %	19.8 %	0.242 %	60.61 %
Water Use	8.89 %	17.9 %	0.365 %	72.9 %
Land Use	9.02 %	9.67 %	0.417 %	80.9 %
Resource Use	20.6 %	13.7 %	0.156 %	65.6 %
**HBBM**				
Climate Change	3.01 %	0.229 %	0.0000477 %	96.8 %
Water Use	0.702 %	0.0444 %	0.0000362 %	99.3 %
Land Use	1.29 %	0.0348 %	0.00000972 %	98.7 %
Resource Use	4.16 %	0.263 %	0.0000432 %	95.6 %

Table S4: Percentage ratios of the ecological impacts of HBBM and DMEM/F12 components from Table S3 on different environmental indicators.

HBBM	Dataset *	Database
**Component**		
**Amino Acids**		
Glycine	RER: glycine production	Ecoinvent
L-Alanine	RER: glycine production	Ecoinvent
L-Arginine hydrochloride		

L-Arginine	Arginine S {FR} U	Agribalyse
L-Asparagine	RER: glycine production	Ecoinvent
L-Asparagine (freebase)	RER: glycine production	Ecoinvent
L-Aspartic acid	RER: glycine production	Ecoinvent
L-Cystine 2HCl	RER: glycine production	Ecoinvent
L-Glutamic Acid	Glutamate {CN} U	Agribalyse
L-Glutamine		
L-Histidine hydrochloride-H_2O		
L-Histidine	Histidine, consumption mix {FR}	Agribalyse
L-Hydroxyproline		
L-Isoleucine	Isoleucine, consumption mix {FR}	Agribalyse
L-Leucine	Leucine, consumption mix {FR} U	Agribalyse
L-Lysine hydrochloride	Lysine solid (HCl), consumption mix {FR}	Agribalyse
L-Methionine	DL-Methionine, processing/RER	Agribalyse
L-Phenylalanine	RER: glycine production	Ecoinvent
L-Proline	RER: glycine production	Ecoinvent
L-Serine	RER: glycine production	Ecoinvent
L-Threonine	L-Threonine, processing/FR U	Agribalyse
L-Tryptophan	Tryptophane, processing/FR U	Agribalyse
L-Tyrosine disodium salt dihydrate	RER: glycine production	Ecoinvent
L-Tyrosine disodium salt	RER: glycine production	Ecoinvent
L-Valine	Valine, processing/FR	Agribalyse
**Vitamins**		
Ascorbic Acid phosphate	GLO: market for ascorbic acid	Ecoinvent
Biotin	GLO: market for citric acid	Ecoinvent
Choline chloride		
D-Calcium pantothenate	RER: market for calcium chloride	Ecoinvent
Folic Acid	GLO: market for ascorbic acid	Ecoinvent
Niacinamide		
Para-Aminobenzoic Acid		
Pyridoxine hydrochloride		
Pyridoxine hydrochloride (206 MW)		
Pyridoxal hydrochloride		
Riboflavin	GLO: market for citric acid	Ecoinvent
Thiamine hydrochloride		
Vitamin B12	GLO: market for citric acid	Ecoinvent
i-Inositol	GLO: market for ascorbic acid	Ecoinvent

Proteins		
AlbuMAX II		
Human Transferrin (Holo)		
Insulin Recombinant Full Chain		
**Inorganic salts**		
Calcium Chloride (CaCl2) (anhyd.)	RER: market for calcium chloride	Ecoinvent
Ferrous sulfate (FeSO47H2O)	RER: market for iron sulfate	Ecoinvent
Magnesium Sulfate (MgSO4) (anhyd.)	GLO: market for magnesium sulfate	Ecoinvent
Potassium Chloride (KCl)	RER: market for potassium chloride	Ecoinvent
Sodium Bicarbonate (NaHCO3)	RER: market for sodium bicarbonate	Ecoinvent
Sodium Chloride (NaCl)	GLO: market for sodium chloride	Ecoinvent
Sodium Phosphate dibasic	GLO: market for disodium phosphate	Ecoinvent
Sodium Phosphate monobasic	RER: market for sodium phosphate	Ecoinvent
Calcium nitrate (Ca(NO3)2 4H2O)	market for calcium nitrate	Ecoinvent
Potassium Nitrate (KNO3)	market for potassium nitrate	Ecoinvent
Zinc sulfate (ZnSO4-7H2O)	RER: market for zinc monosulfate	Ecoinvent
Sodium Selenite		
**Trace elements**		
Ammonium Metavanadate		
Cupric Sulfate	GLO: market for copper sulfate	
Manganous Chloride		
Sodium Selenite		
**Other compounds**		
D-Glucose (Dextrose)	GLO: market for glucose	Ecoinvent
Ethanolamine	market for monoethanolamine	Ecoinvent
Glutathione (reduced)		
Phenol Red	RER: market for phenol	Ecoinvent
Sodium Pyruvate		
HEPES	market for naphthalene sulfonic acid	Ecoinvent

Table S5: The datasets used for the LCA analysis. * Proxy datasets are used based on the recommendation from Blackstone et al. [1]. Empty cells refers to the lack of datasets in the considered databases.

References:

1. Blackstone, N.T.; Pavlova, A.; Trinidad, K.; Nikkhah, A.; Sinke, P.; Heller, M.; DuncanDuggal, J.; Ridoutt, B.; Smetana, S.; Makov, T.; et al. Guidelines for Environmental Life Cycle Assessment of Cultivated Meat 2025.
